# Emerging macrophage-based therapies for cancer: a review of preclinical and clinical advances

**DOI:** 10.3389/fimmu.2025.1679271

**Published:** 2025-10-02

**Authors:** Jan Brancewicz, Paulina Kucharzewska

**Affiliations:** Center of Cellular Immunotherapies, Warsaw University of Life Sciences, Warsaw, Poland

**Keywords:** macrophages, tumor microenvironment, immunotherapy, cancer, cell therapy

## Abstract

Macrophages, the most abundant immune cells in many solid tumors, are no longer viewed solely as accomplices of cancer but as powerful therapeutic allies. This review charts the rapid rise of macrophage-based immunotherapies, from CD47/SIRPα checkpoint blockade and CAR-macrophages to macrophage-drug conjugates (MDCs). We emphasize emerging frontiers - RNA-based reprogramming, epigenetic modulation, small activating RNA and circRNA approaches, and macrophage-derived extracellular vesicles - that are redefining how tumor-associated macrophages can be targeted or harnessed. Distinct from earlier TAM reviews, we integrate outcomes from ongoing and completed clinical trials, highlight therapeutic platforms beyond classical depletion and polarization, and frame macrophages not only as targets but also as delivery vehicles. By spotlighting both innovative strategies and the challenges of moving them into the clinic, we aim to provide a forward-looking guide for researchers and clinicians shaping the next generation of cancer immunotherapy.

## 1 Introduction

The tumor microenvironment (TME) is a dynamic niche that facilitates tumor growth. It comprises immune, stromal, and vascular cells, as well as non-cellular elements such as the extracellular matrix (ECM), signaling molecules, and altered physical and chemical conditions (e.g., hypoxia, acidosis, elevated interstitial pressure), all of which contribute to tumor progression and therapy resistance (1–4). Cancer cells remodel the TME by modifying the ECM, inducing hypoxia and acidity, and releasing signaling molecules and extracellular vesicles (EVs) to influence surrounding cells (5–8). The TME's composition varies across tumor types and evolves with disease progression, becoming increasingly immunosuppressive. Recognizing its critical role, therapeutic strategies have been developed to target immune and stromal components, angiogenesis, and metabolic pathways (9–13). These approaches aim to overcome resistance mechanisms and improve treatment efficacy.

Tumor-associated macrophages (TAMs) are a significant component of the TME and play a central role in cancer progression and treatment (14–16). Certain tumor types can be heavily infiltrated with TAMs, comprising up to 50% of a tumor’s mass (15). Typically, high macrophage infiltration is associated with poor patient prognosis in many types of cancer, such as breast, lung, and gastric cancers (17–19). TAMs constitute a heterogeneous population of myeloid cells. They arise from two main sources: circulating monocytes that infiltrate tumors and differentiate locally, and tissue-resident macrophages (TRMs) that expand *in situ*. The relative contribution of each population varies across tumor types and disease stages. TAMs are most commonly identified by expression of CD68, CD163 (hemoglobin-haptoglobin scavenger receptor), and CD206 (mannose receptor C-type 1, MRC1) markers broadly associated with immunosuppressive and tissue-remodeling functions (20, 21). More recent single-cell studies have revealed additional markers that delineate functionally distinct TAM subsets with prognostic implications. For instance, FOLR2^+^ TAMs (folate receptor β) are enriched in tumors with high CD8^+^ T cell infiltration and correlate with favorable outcomes (22), whereas TREM2^+^ TAMs (triggering receptor expressed on myeloid cells 2) display immunosuppressive transcriptional programs closely related to infiltrating monocytes and are linked to poor prognosis (23). TRMs maintain distinct molecular signatures reflecting their embryonic origin and tissue-specific homeostatic roles. Classical TRM markers include F4/80 (in mice), LYVE1, CD206, and FOLR2 genes associated with vascular maintenance and tissue repair (24). By contrast, CCR2 expression distinguishes monocyte-derived macrophages from TRMs, as CCR2^+^ cells rely on recruitment *via* the CCL2–CCR2 chemokine axis (25). A related but distinct myeloid population in tumors are monocyte-derived dendritic cells (moDCs), which arise from CD14^+^ monocytes under inflammatory conditions and are characterized by high expression of CD11c, HLA-DR (MHC class II), and CD86, while losing CD14 expression during differentiation (26). Functionally, moDCs specialize in antigen cross-presentation and T cell priming, supported by their high expression of costimulatory molecules including CD80, CD83, and ICOSLG (27). By contrast, TAMs tend to adopt immunosuppressive programs that favor tumor progression. Notably, moDCs and TAMs can share overlapping markers such as CD11c and MHC-II, underscoring the need for multi-parameter approaches to resolve their identities within the TME.

Highly plastic and heterogeneous, TAMs influence all stages of tumor development, from initiation to metastasis. Initially, TAMs may exhibit M1-like characteristics, exerting anti-tumor effects through pro-inflammatory cytokine production and cytotoxic activity. However, as tumors progress, TAMs often undergo a shift toward a M2-like phenotype driven by tumor-derived factors, hypoxia, and chronic inflammation (28). M2-polarized TAMs contribute to immune evasion, angiogenesis, ECM remodeling, and metastasis (15, 29). Recent advances in single-cell technologies have revealed substantial TAM heterogeneity defined by distinct transcriptional signatures, spatial localization, and functional programs., challenging traditional M1/M2 classification. Among these, SPP1^+^ TAMs (osteopontin-expressing) are frequently localized to hypoxic or necrotic tumor regions, where they promote ECM remodeling, angiogenesis, and immune exclusion. In head and neck squamous cell carcinoma (HNSCC), drive intravasation and metastasis through secretion of SPP1, CCL18, and CXCL8, with high SPP1^+^ TAM abundance correlating with poor patient prognosis (30, 31). Closely related CCL18^+^ TAMs also exhibit strong immunosuppressive properties, enriched for wound-healing and M2-associated genes such as FN1, CD206, and MMP9, and their presence has been linked to Treg recruitment, epithelial–mesenchymal transition, and unfavorable survival in gastric and other cancers (32–35). Another increasingly recognized subset, TREM2^+^ TAMs, exhibits a lipid-associated, immunosuppressive program; these cells accumulate in hepatocellular carcinoma after transarterial chemoembolisation and suppress CD8^+^ T cell infiltration by downregulating CXCL9 and related chemokines (36, 37). In preclinical models, TREM2 blockade restores intratumoral T cell activity and enhances responses to checkpoint blockade (36). In contrast, FCN1^+^ TAMs represent early-infiltrating cells with monocyte-like inflammatory characteristics. These cells appear upstream of SPP1^+^ and C1Q^+^ TAMs in differentiation pathways, implying their role as plastic precursors that can acquire immunosuppressive functions (33, 38, 39). Collectively, these findings underscore that TAMs encompass a spectrum of functional states shaped by their origin, spatial localization, tumor type, and dynamic cues from the surrounding microenvironment (22, 23). This complexity underscores the need for targeted therapeutic strategies that reprogram TAMs toward anti-tumor phenotypes while minimizing their tumor-supportive functions. Recent years have seen growing research efforts aimed at understanding and modulating macrophage biology to improve cancer treatment.

This review explores the fundamental biology of TAMs and their roles in cancer and provides a comprehensive overview of current macrophage-targeted therapies with the potential to complement and enhance existing cancer treatment strategies. Unlike earlier reviews, our work extends the field by emphasizing clinical trial data, incorporating RNA- and epigenetic-based approaches, and discussing innovative platforms such as macrophage-derived vesicles and drug conjugates.

## 2 Origins and functions of TAMs in cancer

**Figure 1:**
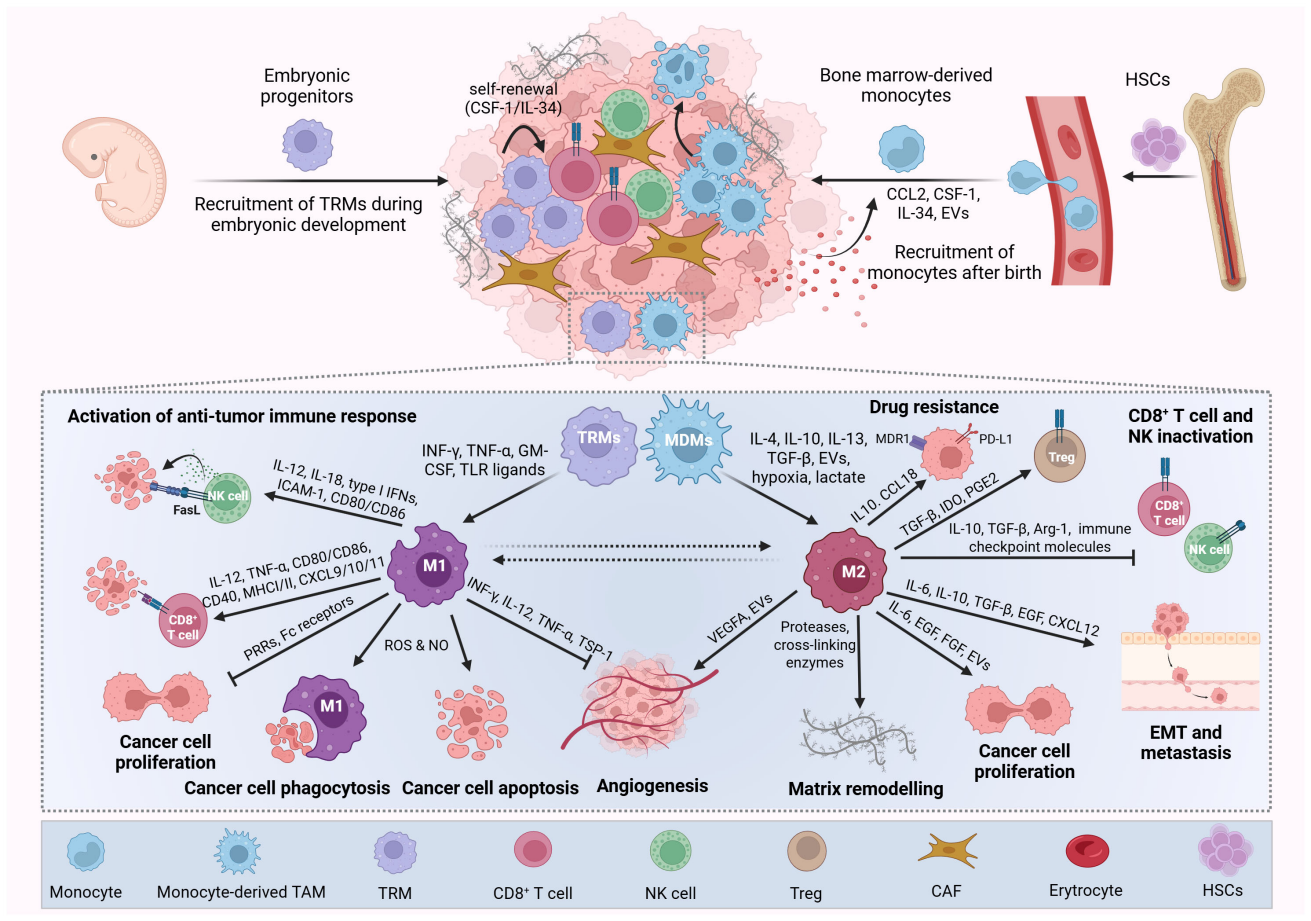
Origin, polarization, and functions of macrophages in cancer. Tumor-associated macrophages (TAMs) arise from two main sources: tissue-resident macrophages (TRMs) and (monocyte-derived macrophages) MDMs. TRMs originate from embryonic yolk sac and fetal liver progenitors during primitive hematopoiesis and persist in adult tissues through self-renewal. In contrast, MDMs are replenished postnatally by circulating monocytes derived from hematopoietic stem cells in the bone marrow. In the tumor microenvironment, cancer and stromal cells secrete cytokines and chemokines that promote monocyte recruitment and differentiation into MDMs. Together, TRMs and MDMs constitute a heterogeneous and often dominant immune cell population within many tumors, contributing significantly to cancer progression. TAMs are broadly classified into classically activated (M1-like) and alternatively activated (M2-like) phenotypes. M1-like TAMs are induced by pro-inflammatory cytokines (e.g., IFN-γ, TNF-α, GM-CSF, TLR ligands) and exert antitumor effects by promoting immune responses, inhibiting tumor proliferation and angiogenesis, and inducing cancer cell death and phagocytosis. Conversely, M2-like TAMs are polarized by factors such as IL-4, IL-10, IL-13, TGF-β, EVs, hypoxia, and lactate and support tumor progression by enhancing cancer cell proliferation, angiogenesis, immune evasion, multidrug resistance, invasion, and metastasis, as well as suppressing cytotoxic immune cells (e.g., CD8^+^ T cells, NK cells) and recruiting regulatory T cells (Tregs). Picture created using BioRender.

In the context of cancer, two primary macrophage populations are recognized: monocyte-derived macrophages (MDMs) and TRMs (25, 40) (**Figure 1**). Emerging evidence also highlights the spleen as an extramedullary reservoir for myeloid precursors in cancer models (41). This complex origin contributes significantly to macrophage heterogeneity and functional diversity in the TME. The relative contribution of TRMs and MDMs varies by tumor type and stage, with both populations often coexisting within the same tumor. This coexistence has been demonstrated in lung adenocarcinoma (42), glioblastoma (GBM) (43), hepatocellular carcinoma (44–48), pancreatic ductal adenocarcinoma (PDAC) (49, 50), breast cancer (22, 51), ovarian cancer (52), and colorectal cancer (53).

### 2.1 Monocyte-derived macrophages in cancer

MDMs represent a critical component of the TME, playing multifaceted roles in cancer progression, metastasis, and therapeutic resistance (54, 55). They originate primarily from circulating monocytes recruited to the tumor site through complex chemotactic mechanisms that are orchestrated by chemokines and growth factors secreted by tumor cells and the TME stromal components (56). The signaling pathway involving the chemokine (C-C motif) ligand 2 (CCL2) (also referred to as monocyte chemoattractant protein 1, MCP1) and its receptor CCR2, has been identified as a key driver mediating this process in most solid tumors. Tumor and stromal cells secrete CCL2, which attracts CCR2-expressing inflammatory monocytes that subsequently differentiate into TAMs within the TME (57, 58). Additional monocyte recruitment pathways include the following ligand-receptor interactions: colony-stimulating factor-1 (CSF-1)/CSF-1 receptor (CSF-1R) (59, 60), IL-34/CSF-1R (61), CX3C chemokine ligand 1 (CX3CL1) (also known as fractaline)/CX3CR1 (62, 63), CCL3/CCR1 (64), CCL3/CCR5 (65), CCL5/CCR5 (65, 66), CCL20/CCR6 (67), and vascular endothelial growth factor A (VEGF-A)/VEGFR1 (68). The CSF-1/CSF-1R and IL-34/CSF-1R axes are particularly important as they contribute to both monocyte recruitment and M2 polarization while supporting the self-renewal of TRMs (69).

EVs are additional modulators of monocyte recruitment and function in cancer. They promote chemotaxis by delivering cargos that activate chemokine-receptor signaling. For example, LC3^+^ EVs from breast cancer activate lung fibroblasts *via* TLR2–MyD88–NF-κB signaling, promoting monocyte recruitment and T cell suppression within pre-metastatic niches (70). Lung macrophages internalizing EVs containing complement C3 increase CCL2 and CXCL1 secretion, enhancing recruitment of TAMs and polymorphonuclear myeloid-derived suppressor cells (71). Cytokines like CCL2 may also bind exosomal proteoglycans, promoting CCR^+^ monocyte recruitment and activation (72). Additionally, EVs from colon, lung, and pancreatic tumors can transfer CCR6 to monocytes, increasing their responsiveness to CCL20 (73). Beyond recruitment, tumor-derived EVs drive monocyte differentiation into immunosuppressive phenotypes that support tumor immune evasion (74, 75). It is important to distinguish these tumor-derived vesicles, which promote monocyte recruitment and immunosuppression, from macrophage-derived EVs being developed as therapeutic delivery systems (discussed in Section 8.4).

Hypoxia and elevated lactate levels in TME significantly influence monocyte and MDMs recruitment and function. Hypoxic stress induces chemoattractants such as VEGF-A, endothelin-2, CCL26, and CXCL12, guiding TAMs to low-oxygen tumor regions (56, 76, 77). Recruited TAMs undergo hypoxia inducible factor-1α (HIF-1α) and HIF-2α-driven reprogramming, enhancing angiogenic activity while reducing motility (78). In PDAC, lactate induces K63 lactylation of endosulfine alpha, activating STAT3-CCL2 signaling and promoting TAM accumulation and immunosuppression (79). Therapies also modulate TAM recruitment: radiation increases CXCL12 at invasive margins, and chemotherapy elevates CXCL12 near vasculature, both enhancing TAM infiltration (80). Additionally, IL-34 upregulation in refractory melanoma correlates with CD163^+^ macrophage enrichment (81, 82).

Upon tumor infiltration, circulating monocytes differentiate into MDMs *via* pathways regulated by CSF-1 and IL-34 signaling through CSF-1R (83). Under early onset of cancer or inflammatory conditions (e.g., IFN-γ), MDMs can adopt an anti-tumor phenotype. However, in established tumors, MDMs are typically driven toward an immunosuppressive state by interleukin-4 (IL-4), IL-10, transforming growth factor β (TGF-β), and hypoxia (84) (explored in Section 3).

Although monocyte-derived TAMs may initially exert anti-tumor effects through several mechanisms such as phagocytosis, antibody-dependent cellular cytotoxicity, tumor necrosis, and activation of native and adaptive immune responses (85, 86), the evolving TME reprograms these cells to drive tumor development and progression. In this state, monocyte-derived TAMs support tumor progression by secreting growth factors that promote cancer cell proliferation and by stimulating angiogenesis, particularly in hypoxic regions (87, 88). They also contribute to immune evasion by suppressing T cell and natural killer cell activity and recruiting immunosuppressive Tregs (29, 89, 90). Furthermore, TAMs facilitate metastasis through ECM degradation, induction of epithelial-mesenchymal transition (EMT), and establishment of pre-metastatic niches in distant tissues (91). Collectively, these pro-tumor activities of monocyte-derived TAMs compromise the efficacy of chemotherapy, radiotherapy, and immunotherapy (92).

Diverse pro-tumor functions of monocyte-derived TAMs make them critical targets for developing therapies aimed at limiting tumor growth, overcoming treatment resistance, and preventing metastasis (93). Strategies targeting monocyte-derived TAMs focus primarily on blocking their recruitment or reprogramming them toward pro-inflammatory states (Section 5). Combining these approaches offers potential to suppress tumor growth and improve therapeutic efficacy.

### 2.2 Tissue-resident macrophages in cancer

TRMs originate during embryogenesis from yolk sac and fetal liver progenitors, seeding organs where they self‐renew throughout life. In organs such as the brain (microglia), liver (Kupffer cells), and lungs (alveolar macrophages, AMs), TRMs constitute the dominant macrophage population that performs specialized homeostatic functions (94, 95). TRMs constitute a highly heterogeneous group of cells expressing distinct markers depending on their tissue of origin (96, 97). Emerging evidence suggests that TRMs are essential components of the TME in various types of malignancies. Unlike MDMs, which require continuous bone marrow replenishment, TRMs are sustained in the TME through tumor-derived cytokines such as CSF-1 and IL-34, which activate CSF-1R signaling to promote their survival and proliferation (69, 98). Cytokines and growth factors in the TME reprogram TRMs to adopt pro-tumorigenic phenotypes, including immune suppression, angiogenesis, and stromal remodeling, while retaining lineage-specific traits that differ across cancer types (42, 99).

TRMs represent promising targets for cancer therapy due to their involvement in tumor development and progression. However, their embryonic origin, capacity for self-renewal, and tissue-specific maintenance present therapeutic challenges. Unlike monocyte-derived TAMs, TRMs cannot be effectively targeted through inhibition of recruitment pathways alone. Potential strategies may include selective depletion using tissue-specific markers such as CD163 (in the omentum) or LYVE-1 (in breast cancer), inhibition of CSF-1/CSF-1R signaling, and reprogramming TRMs to restore or enhance their anti-tumor functions while preserving their physiological roles (Section 5).

#### 2.2.1 TRMs in lung cancer

In lung cancer, both TRMs and MDMs contribute to the pool of TAMs. Interstitial macrophages support tumor growth, while recruited macrophages drive tumor spread (100). The second type of lung TRMs, AMs, also contribute to tumor progression, but in early lesions (101). Over time, AMs are gradually replaced by MDMs, further shaping the TME (102). In early-stage lung cancer, AMs create a pro-tumorigenic niche by promoting activin A-dependent lung cancer cell proliferation and enhancing tumor invasiveness *via* EMT by upregulating TWIST1 and suppressing E-cadherin (42, 103). A specific AM subset, S100a4^+^ AM, drives early malignant transformation by enhancing lipid metabolism and angiogenesis, correlating with poor prognosis and epithelial plasticity (104). AMs also establish an immunosuppressive niche by inducing Treg responses, shielding tumor cells from CD8^+^ T cell attacks (42, 101, 105, 106), and adopting an immunosuppressive phenotype marked by reduced cytokine production, MHCII expression, and co-stimulatory molecules essential for adaptive immunity (102, 107). Beyond primary tumor growth, AMs facilitate lung metastasis. β-catenin activation in AMs fuels metastasis *via* a tumor necrosis factor-α (TNF-α)-driven inflammatory program (108), while in metastatic hepatocellular carcinoma, 5-LOX-expressing AMs secrete leukotriene B4 (LTB4) to support cancer proliferation (109). Additionally, lung macrophages foster an immune-evasive pre-metastatic niche of breast cancer by upregulating programmed death-ligand 1 (PD-L1), which correlates with increased Treg infiltration (106), and by suppressing dendritic cell maturation and T cell function through complement C5a signaling, promoting accumulation of immunosuppressive AMs in premetastatic lung areas (110). In lung adenocarcinoma, tumor-derived IL-4 and IL-13 activate STAT6 signaling in TRMs, promoting their transition into pro-fibrotic cells that deposit collagen and recruit cancer-associated fibroblasts (111).

#### 2.2.2 TRMs in GBM

TAMs play a crucial role in the development and progression of GBM (112, 113). These cells consist of both microglia (TRMs), the resident immune cells of the central nervous system, and MDMs (114). Monocyte-derived TAMs account for approximately 85% of GBM-associated TAMs, while microglia make up about 15% (115). Although MDMs predominate in the GBM TME, microglia are involved in various pro-tumorigenic processes. GBM cells employ multiple mechanisms to recruit and reprogram microglia. GBM-derived factors such as CSF-1 (116), glial cell-derived neurotrophic factor (GDNF) (117), CCL2 (118), MIC-1 (119), S100A8 (120), TLR-2 ligands (121), CXCL12 (77), versican (122), and Wnt3a activating the Wnt/β-catenin pathway (123, 124), play pivotal roles in this process. CSF-1 is essential for microglial survival and proliferation, and its increased expression in GBM correlates with enhanced microglial infiltration and tumor progression (125–127). CCL2 is another important chemokine that promotes the infiltration of microglia, and its expression level is highly correlated with the grade of glioma (118, 128). Moreover, recruitment and activation of the pro-tumorigenic phenotypes in microglia can be driven by factors released from GBM or stromal cells residing in the hypoxic niches and glioma stem cells (129, 130). Emerging evidence confirms that GBM-derived EVs also play a critical role in reprogramming microglia in the GBM TME by delivering miRNA cargo (131, 132). Additionally, GBM cells upregulate PD-L1 expression on microglia or secrete PD-L1 that activates PD-1 positive microglia, triggering anti-inflammatory (M2) macrophage subtype and promoting an immunosuppressive TME (133, 134).

#### 2.2.3 TRMs in pancreatic cancer

In PDAC, both MDMs and pancreas-resident macrophages are essential components of the TME and contribute to tumor progression (135). However, whereas MDMs are more potent at antigen presentation, embryonically derived TAMs exhibit a pro-fibrotic phenotype, indicating their role in remodeling of the ECM and fibrosis (49, 136). Prolactin seems to be one of the factors driving the pro-fibrotic activity of pancreas-resident macrophages (137). Additionally, TRMs are strongly associated with poor clinical outcomes and chemoresistance of PDAC (138). TRMs self-renew locally under the influence of CSF-1 secreted by cancer cells and cancer-associated fibroblasts, independent of CCR2^+^ monocyte recruitment. This self-renewal capacity enables TRMs to persist even when monocyte-derived TAMs are depleted, contributing to tumor progression (49, 139).

#### 2.2.4 TRMs in ovarian cancer

TAMs account for over 50% of the cellular population in the ovarian cancer TME, including in ascitic fluid from patients with peritoneal metastases (140, 141). Distinct tissue-resident macrophage subsets, particularly large peritoneal macrophages (LPMs) and omental macrophages, play critical tumor-promoting roles within the peritoneal cavity and omentum, common sites of ovarian cancer dissemination (141).

The omentum, a fatty tissue layer in the peritoneal cavity, contains immune aggregates called milky spots, which harbor macrophages, T and B cells, and vasculature that facilitate metastatic colonization. Among omental macrophages, embryonically derived CD163^+^ Tim4^+^ TRMs are key players in forming pre-metastatic niches (141). These macrophages secrete chemokines such as CCL6/CCL23 to recruit tumor cells *via* CCR1 (142). They also promote EMT and stemness through IL-6, erythropoietin, and prolactin signaling. Their depletion significantly suppresses tumor progression (52). Ovarian cancer cells reprogram omental macrophages through EVs enriched in laminin and proteins like eIF4E, inducing M2 polarization, PD-L1 upregulation, and secretion of CXCL5 and CCL2 (143). Additionally, hyaluronic acid secreted by tumor cells triggers cholesterol efflux in macrophages, activating PPAR-γ and reinforcing immunosuppressive M2-like functions by suppressing antigen presentation and enhancing IL-4 signaling (144).

LPMs, derived from embryonic progenitors and maintained through self-renewal, infiltrate early ovarian tumors and contribute to tumor growth and metastasis (145, 146). However, not all resident macrophages uniformly promote the disease. Some Tim4^+^ LPMs can capture and cross-present tumor antigens, potentially contributing to initial immune surveillance (147).

#### 2.2.5 TRMs in breast cancer

Mammary gland TRMs (MGTRMs) contribute to breast cancer development and progression by modulating the TME. Derived from embryonic yolk sac precursors, MGTRMs form self-renewing populations established during mammary gland development (148). In healthy tissue, they reside in the adipose stroma and near ductal epithelium, supporting homeostasis through ECM remodeling, apoptotic cell clearance, and ductal morphogenesis (24, 149, 150). Distinct MGTRM subpopulations exist within the mammary microenvironment, including CXCR4^+^, LYVE-1^+^, and FOLR2^+^ macrophages, each characterized by different surface markers and functional specializations.

In breast cancer, CXCR4^+^ ductal macrophages promote tumor-initiating cells (TICs) by fostering stem-like niches and facilitating immune evasion and EMT (151). In triple-negative breast cancer, MGTRMs are the predominant stromal population early in disease progression and are critical for tumor growth, recurrence, and chemoresistance (51). A key pathway involves IL-17A-induced osteopontin expression *via* CEBPβ in cancer cells, which activates LYVE-1 on MGTRMs, promoting immunosuppressive expansion through the JNK/c-Jun pathway. Osteopontin also recruits LYVE-1^-^ MDMs *via* α4β1 integrin, further enriching the immunosuppressive TME. Targeting this axis enhances anti-PD-L1 therapy response, identifying LYVE-1^+^ MGTRMs as noteworthy therapeutic targets in breast cancer (152).

In contrast to LYVE^+^ MGTMRs, FOLR2^+^ MGTRMs support anti-tumor immunity by co-localizing with CD8^+^ T cells and enhancing their activation through the CXCL9-CXCR3 axis. This interaction is linked to increased tumor apoptosis, reduced invasion, and improved patient outcomes (153).

## 3 Polarization and phenotypic diversity of TAMs

Within the TME, TAMs exist along a functional spectrum between two extremes: the proinflammatory, anti-tumor M1 phenotype and the anti-inflammatory, pro-tumor M2 phenotype. The dynamic polarization of TAMs toward either phenotype plays a crucial role in tumor progression, significantly influencing cancer prognosis and therapeutic outcomes. In the early stages of tumor development, M1 macrophages often dominate, contributing to an anti-tumor immune response (85, 96). However, as the tumor evolves, there is a progressive shift toward M2-like macrophages, which support tumor growth, immune suppression, and metastasis (96, 134). This phenotypic switch is driven by various factors in the TME, including chronic inflammation, persistent hypoxia, nutrient deprivation, and an altered cytokine milieu (96). Nevertheless, the classical M1/M2 dichotomy oversimplifies the complex biology of TAMs. Emerging evidence from single-cell analyses reveals that TAMs do not exist as discrete populations but rather display a continuum of activation states. These cells often co-express markers of both M1 and M2 phenotypes, with their functional profiles shaped by dynamic interactions with tumor cells, stromal components, and metabolic signals in the TME.

### 3.1 M1 (proinflammatory) macrophages

M1 macrophage polarization plays a vital role in anti-tumor immunity by fostering inflammation and tumor destruction (85, 86, 154). The factors promoting M1 macrophage polarization in tumors include IFN-γ, Toll-like receptor (TLR) ligands, granulocyte-macrophage colony-stimulating factor (GM-CSF), and TNF-α, which activate signaling pathways such as STAT1, NF-κB, and IRF5/8. These cytokines and signals come from immune cells like Th1 cells, CD8^+^ T cells, NK cells, and dendritic cells (140). Environmental factors such as iron overload, oxidative stress, and d-lactate also promote M1 polarization (155–157).

M1 macrophages exhibit diverse molecular signatures that reflect their pro-inflammatory status, including the expression of surface (HLA-DR, CD86, CD80, MHC-II) and functional markers: pro-inflammatory cytokines (TNF-α, IL-12, IL-23, IL-6), chemokines (CXCL9, CXCL10, CXCL11), nitric oxide synthase (iNOS), matrix metalloproteinases (MMP1, MMP9) and phosphorylated STAT1 transcription factor (86). Metabolically, M1 macrophages shift to aerobic glycolysis and rely on HIF-1α to sustain their inflammatory functions (158). M1 macrophages are crucial for initiating inflammatory responses and exhibit potent tumoricidal activities, including robust phagocytosis of tumor cells, producing pro-inflammatory cytokines (e.g., TNF-α, IL-1β, IL-6), reactive oxygen species (ROS), and nitric oxide (NO) (145, 146). Additionally, they can function as antigen-presenting cells (APCs) within solid tumors. TAMs express MHC class II and costimulatory molecules such as CD80 and CD86, enabling them to process and present tumor-derived antigens to CD4^+^ T cells (29). Although this function is generally less efficient than that of DCs, TAM-mediated antigen presentation contributes to shaping the local T cell response, particularly in contexts where DC numbers are limited. Mechanistically, antigen presentation by TAMs has been shown to influence the differentiation and exhaustion of tumor-infiltrating T cells: for example, TAM antigen presentation can drive progenitor-exhausted T cells toward a terminally exhausted state, with direct consequences for responsiveness to immune checkpoint blockade (159). Conversely, when TAMs present antigen in an immunostimulatory context (e.g., with appropriate costimulation or innate activation), they can support local T cell proliferation and effector function (160). However, this process is often counterbalanced by the immunosuppressive programming of TAMs, which can upregulate inhibitory ligands such as PD-L1 or secrete cytokines like IL-10 that limit effective T cell responses. Together, these findings position TAMs as key local determinants of intratumoral T cell fate and suggest that therapeutically reprogramming TAM antigen-presentation phenotypes could shift the intratumoral balance from terminal exhaustion toward sustained, checkpoint-responsive antitumor immunity.

### 3.2 M2 (anti-inflammatory) macrophages

M2 macrophages are a key immunosuppressive and pro-tumorigenic subset of TAMs within the TME (87). M2 macrophages arise in response to a complex interplay of cytokines, metabolic factors, tumor-derived signals, and modified ECM, that collectively reprogram macrophage phenotype and function through specific molecular pathways (161). The initiation of M2 polarization is primarily driven by cytokines, CSF-1 and IL-34, and chemokines, CCL2 and CCL5, that facilitate TAM recruitment into the TME and transition to M2 TAMs. Other important factors include anti-inflammatory cytokines such as IL-4, IL-10, IL-13, and TGF-β (119, 162). Additional factors include tumor cell-derived EVs enriched with microRNAs (e.g., miR-21, miR-138-5p, miR-106a-5p) and proteins that directly reprogram macrophages into M2 states (90, 163, 164). In parallel, the unequal distribution of oxygen and nutrients within the TME creates distinct microniches that influence macrophage polarization. Macrophages located near perfused vessel areas, where oxygen, glucose, and glutamine levels are high, tend to polarize toward the M1 phenotype. Conversely, macrophages residing in poorly vascularized regions characterized by chronic hypoxia and metabolic byproducts like lactate and succinate, which accumulate under hypoxic and glycolytic conditions, stabilize HIF-1α and reinforce M2 (78, 165–167). Changes in ECM stiffness and remodeling exert mechanical stress on macrophages *via* mechanoreceptors, biasing them toward M2 polarization. These structural changes in the TME contribute to the persistence of pro-tumor macrophage phenotypes (168). Understanding these complex polarization mechanisms has significant implications for developing strategies to reprogram TAMs toward anti-tumor phenotypes as a therapeutic approach.

M2 TAMs display various markers that provide them with pro-tumorigenic activities, including CD163, CD204, CD206, CD200R, CD209, CD301, CCR2, CSF-1R, and PD-L2. CD206 has emerged as a particularly reliable marker that faithfully reflects M2 macrophage abundance and is significantly upregulated in various cancer types (21, 169). Functional markers of M2 TAMs include anti-inflammatory cytokines (IL-10, TGF-β), chemokines (CCL17, CCL18, CCL22, CCL24), arginase-1, and growth factors, e.g., epidermal growth factor (EGF), VEGF, and platelet-derived growth factor-β (PDGF-β) (161, 170). M2 TAMs contribute to an immunosuppressive microenvironment that fosters tumor growth. They promote angiogenesis by secreting VEGF and other pro-angiogenic factors, facilitating tumor vascularization and progression. Moreover, M2 TAMs enhance ECM remodeling and induce EMT, thereby accelerating cancer dissemination (171). Their abundance is frequently associated with poor prognosis across various cancer types (172–174).

## 4 Macrophages as biomarkers in cancer

TAMs are increasingly recognized as valuable biomarkers for cancer prognosis and treatment response. Their density and phenotype significantly influence clinical outcomes, although these effects vary depending on the cancer’s type. High infiltration of CD68^+^ TAMs, as measured by immunohistochemistry, generally correlates with poor prognosis in several malignancies, including breast, lung, and ovarian cancers (175–177). In contrast, patients diagnosed with colorectal cancer often demonstrate improved outcomes with increased TAM presence, particularly when accompanied by high T cell infiltration, underscoring the context-dependent nature of macrophage function (178).

The M1/M2 polarization ratio provides further prognostic value, with a higher ratio indicating a more favorable, pro-inflammatory immune environment (179). In parallel, non-invasive imaging techniques such as magnetic resonance imaging with macrophage-specific contrast agents enable non-invasive assessment of TAM distribution and response to therapy, providing real-time biomarkers for monitoring treatment efficacy (180).

In certain cancers, the differential expression and histologic distribution of TAM markers such as CD86 and CD163 provide a more accurate prediction of patient survival than the overall number of TAMs (181). In colorectal cancer, elevated levels of CD86^+^ and CD68^+^CD86^+^ TAMs, alongside reduced levels of CD163^+^ and CD68^+^CD163^+^ TAMs, are linked to better overall survival (182). Similarly, increased infiltration of CD163^+^ TAMs has been linked to poorer outcomes in other malignancies, including gastric cancer (183), clear cell renal cell carcinoma (184), head and neck squamous cell carcinoma (181), and non-small cell lung cancer (NSCLC) (88). In NSCLC, survival analysis showed that lymph node metastasis, along with high densities of CD68^+^ and CD204^+^ TAMs in the tumor stroma, but not in tumor islets or alveolar space, were independent predictors of poor prognosis (185). These findings suggest that the characterization of TAM phenotype and spatial location within the tumor may offer more precise prognostic insights than total TAM counts alone.

Single-cell RNA sequencing has identified four distinct TAM subpopulations with unique transcriptional profiles and clinical relevance. SPP1^+^ TAMs, expressing genes like *secreted phosphoprotein 1* (*SPP1*), *macrophage receptor with collagenous structure* (*MARCO*), *VEGFA*, and *fibronectin 1* (*FN1*), are prevalent across cancers and linked to hypoxia, metastasis, angiogenesis, and poor prognosis (186). C1Q^+^ TAMs, characterized by *A/B/C-chain polypeptide of the complement component C1q* (*C1QA/B/C*), *PD-L1*, *PD-L2*, and *triggering receptor expressed on myeloid cells 2* (*TREM2*) expression, play roles in antigen presentation and immune regulation, and are found in colorectal, lung, and cervical cancers (39). Their impact on prognosis varies: in cervical and pancreatic cancers, C1QC^+^ TAMs correlate with better outcomes, while TREM2^+^ TAMs in GBM and esophageal squamous cell carcinoma are associated with poor survival (187). FCN1^+^ TAMs, characterized by high expression of ficolin-1 (FCN1), are monocyte-derived, pro-inflammatory, and antigen-presenting TAMs (33). High FCN1 expression has been associated with better survival in acute myeloid leukemia (AML), indicating its utility as a prognostic biomarker. CCL18^+^ TAM subpopulation is a terminally differentiated subset of TAMs characterized by high expression of the chemokine CCL18. These macrophages display M2-like features, and are implicated in promoting tumor progression, metastasis, therapy resistance, and immune evasion across multiple solid tumor types (188). Paradoxically, higher tumor-infiltrating CCL18^+^ TAMs correlate with better survival in NSCLC and gastric cancer, indicating context-dependent roles across cancers (189).

TAMs also hold predictive value in the context of immunotherapy. In NSCLC, TAM-related markers such as CSF-1R and hematopoietic cell signal transducer (HCST) have demonstrated superior predictive power compared to PD-L1, showing strong correlations with both PD-L1 expression and CD8^+^ T cell infiltration (190). In esophageal squamous cell carcinoma, high infiltration of TREM2^+^ TAMs not only serves as a prognostic biomarker but is also associated with resistance to immune checkpoint blockade (23). As a result, TAM-related biomarkers are increasingly being incorporated into diagnostic strategies for immunotherapy, with the potential to improve patient stratification and enhance clinical outcomes.

## 5 Targeting and using macrophages in therapy

**Figure 2:**
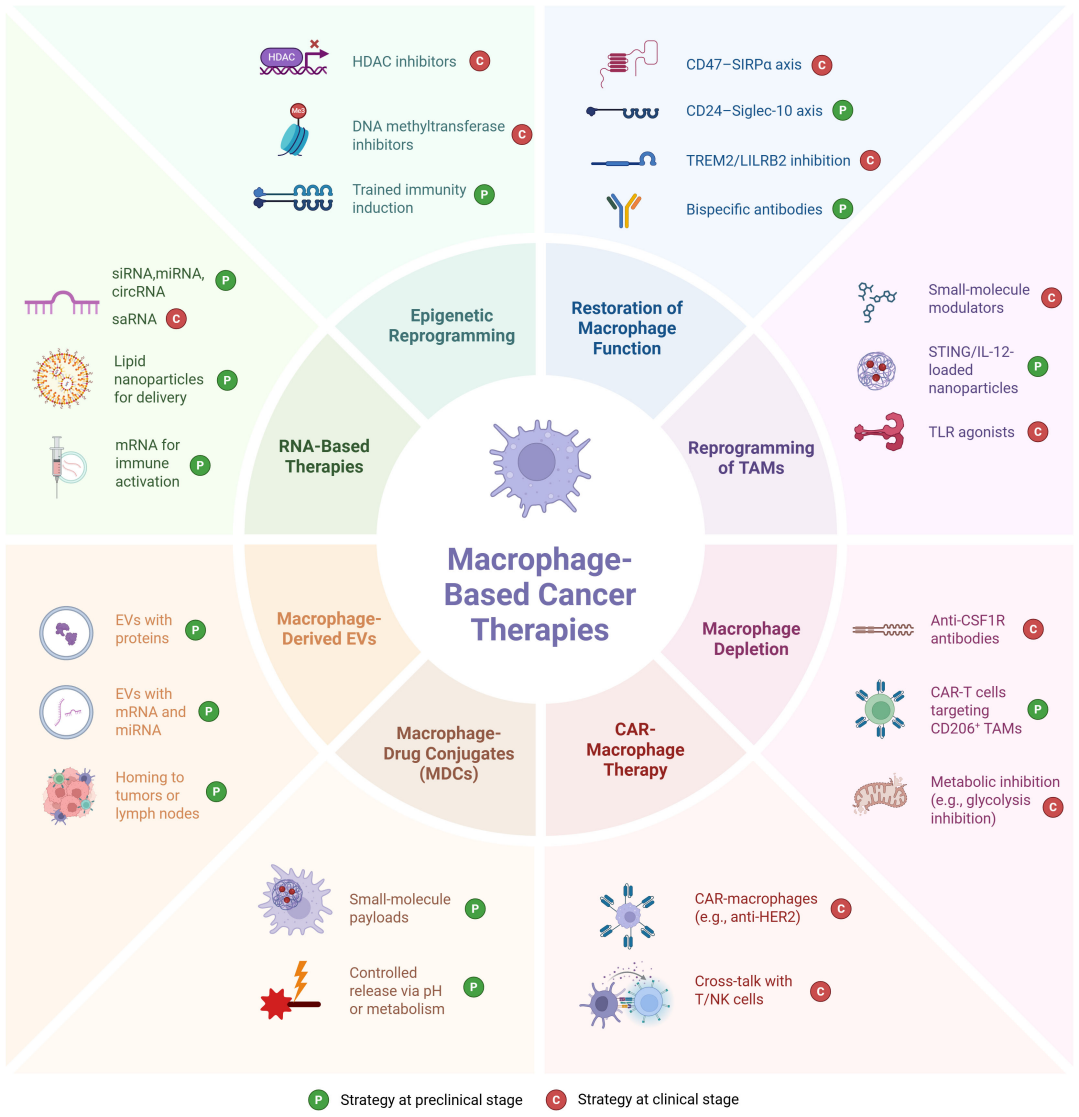
Overview of eight therapeutic strategies for targeting tumor-associated macrophages in the tumor microenvironment. Clinical status: CD47–SIRPα (magrolimab, evorpacept and others in trials) (191), TREM2 (PY314 ± pembrolizumab, Ph1) (192), LILRB2 (IO-108, Ph1) (193), PI3Kγ modulators (eganelisib/IPI-549 in Ph1/2 combos) (194), TLR agonists (e.g., imiquimod approved for sBCC; TLR9 agonist tilsotolimod tested in Ph3 melanoma) (195), CSF1R inhibitors (emactuzumab and others in trials; pexidartinib approved for tenosynovial giant cell tumor, TGCT) (54), CAR-macrophages (FIH Ph1 CT-0508) (196) and cross-talk with T/NK cells (mechanistic outcome of CAR-M) (197), HDAC inhibitors (resminostat in Ph1/2 solid tumor trials (198)), and DNA methyltransferase inhibitors (199) have human oncology trials; CD24–Siglec-10 (oncology use largely preclinical; CD24Fc tested in non-oncology) (200, 201), macrophage-engaging bispecifics (202), IL-12/STING nanoparticles (STING and IL-12 have clinical trials, but NP-loaded macrophage-targeted formats remain preclinical) (203), TAM-depleting CAR-T (204), macrophage-derived EVs (205), MDCs (206), most TAM-directed RNA (207) [except saRNA MTL-CEBPA (208)], and trained immunity induction (209) remain preclinical. Picture created using BioRender.

Macrophages are essential components of the TME, where they can either promote or inhibit tumor progression depending on their phenotype. This dual nature makes macrophages both valuable therapeutic tools and critical targets in cancer therapy. Modern therapeutic strategies leverage their plasticity and functional diversity, focusing on reprogramming macrophages to adopt anti-tumor phenotypes, depleting TAMs, or restoring their intrinsic anti-cancer functions. While significant advances have been made in understanding and targeting macrophages, these therapies remain complex due to the dynamic nature of macrophage phenotypes and their interactions within the TME. The most promising therapeutic strategies are summarized in **Figure 2**.

### 5.1 Restoration of macrophage function

Restoring the anti-tumor functions of TAMs involves exploiting their plasticity to reprogram them from an M2-like (tumor-promoting) phenotype to an M1-like (pro-inflammatory, tumoricidal) state. M1 macrophages enhance anti-tumor immune responses through cytokine production and the recruitment of immune cells, while M2 macrophages promote immunosuppression, angiogenesis, and metastasis.

#### 5.1.1 CD47/SIRPα blockade

One prospective approach to reprogram TAMs is blocking the CD47/SIRPα axis. Tumor cells often exploit CD47, a "don't eat me" signal, to evade macrophage-mediated phagocytosis. The CD47/SIRPα blockade restores macrophages' ability to recognize and engulf cancer cells, leading to increased apoptosis and activation of antibody-dependent cellular cytotoxicity. This strategy also enhances the anti-tumor activity of NK cells, creating a synergistic immune response (210).

Multiple clinical trials have demonstrated the therapeutic potential of CD47 blockade in cancer treatment. Magrolimab, an anti-CD47 monoclonal antibody, has shown promising efficacy in combination therapies. In a Phase 1b trial for higher-risk myelodysplastic syndromes (HR-MDS), the combination of magrolimab and azacitidine resulted in a 33% complete remission rate and a 75% overall response rate (ORR) (211).

Similarly, evorpacept (ALX148), a high-affinity CD47 blocker, has shown efficacy in solid tumors. In a Phase 2 trial for HER2-positive gastric cancer, the combination of evorpacept with trastuzumab and chemotherapy led to a 52% ORR, significantly improving outcomes compared to the 22% ORR observed with standard treatment (212).

These findings underscore the clinical utility of CD47 blockade across multiple cancer types, particularly in combination with other immunotherapies. However, challenges remain in CD47-targeted therapies, including on-target effects on red blood cells, leading to mild anemia in some patients. Strategies such as preferentially targeting tumor-overexpressed CD47 variants or combining CD47 blockade with tumor-specific antibody opsonization are being explored to reduce off-target toxicity while maximizing therapeutic benefits (210).

Other macrophage immune checkpoints are also under investigation. Anti-SIRPα agents, such as TTI-621, are being tested clinically to enhance macrophage-mediated phagocytosis by blocking the SIRPα/CD47 axis, an alternative “don’t eat me” signal used by tumors (213).

#### 5.1.2 CD24/Siglec-10 blockade

Blockade of the CD24/Siglec-10 axis is increasingly recognized as an effective method to restore macrophage-mediated tumor clearance. CD24, a glycoprotein overexpressed on cancer cells, interacts with Siglec-10, an inhibitory receptor on macrophages, to suppress phagocytosis and promote immune evasion. Inhibiting this interaction enhances macrophage-mediated tumor clearance and reduces tumor growth *in vivo* (214).

Recent preclinical studies have further validated the therapeutic potential of CD24 blockade. IMM47, an IgG1 monoclonal antibody targeting CD24, has demonstrated significant tumor reduction in mouse models by enhancing macrophage phagocytosis and inducing both antibody-dependent cellular cytotoxicity and antibody-dependent cellular phagocytosis. Ongoing research is evaluating whether combining IMM47 with anti-PD-1 checkpoint inhibitors can further boost anti-tumor immune responses (215, 216).

Additionally, there is growing interest in dual blockade of CD47 and CD24 as a potential synergistic strategy to overcome tumor immune evasion (214). While no clinical trials specifically targeting both checkpoints together have been reported yet, preclinical findings suggest that this dual inhibition could enhance macrophage activation and improve therapeutic efficacy. Continued research and translational studies will be crucial in determining the viability of CD24-targeted therapies in combination with existing immunotherapies.

#### 5.1.3 TREM2 blockade

Recent research has identified TREM2 as an important macrophage checkpoint and a promising immunotherapy target. TREM2^+^ TAMs are enriched in immunotherapy-resistant tumors and exhibit strong immunosuppressive properties, reducing CD8^+^ T cell infiltration and promoting an immunologically “cold” tumor environment (217).

Blockade of TREM2 models led to TAM depletion, enhanced antigen presentation, and increased T cell infiltration into tumors. This resulted in significant tumor reduction, especially when TREM2 blockade was combined with immune checkpoint inhibitors (ICIs). These findings have led to the clinical development of PY314, a first-in-class anti-TREM2 monoclonal antibody, which is currently in Phase 1 clinical trials in combination with pembrolizumab for advanced solid tumors. Early data indicate acceptable safety and pharmacologic activity, with ongoing trials aiming to establish efficacy (192).

Given that TREM2^+^ TAMs correlate with checkpoint inhibitor resistance, targeting this axis could convert non-responsive tumors into ICI-sensitive tumors, making TREM2 blockade a valuable strategy in immunotherapy combinations.

#### 5.1.4 LILRB2/ILT4 blockade

In addition to these macrophage immune checkpoints, LILRB2 (also known as ILT4) is considered another inhibitory receptor highly expressed on CD163^+^ TAMs. LILRB2 suppresses macrophage activation by inhibiting pro-inflammatory signaling pathways, promoting an immunosuppressive TME. To counteract this effect, companies are developing anti-LILRB2/ILT4 antibodies, with JTX-8064 being one of the lead candidates. Preclinical studies have shown that blocking LILRB2 reprograms human macrophages to a stimulatory phenotype, enhances antigen presentation, and promotes T cell activation. The INNATE clinical trial is currently evaluating JTX-8064 in cancer patients, where initial pharmacodynamic data from *ex vivo* tumor cultures suggest on-target activity (218).

#### 5.1.5 Macrophage-engaging bispecific antibodies

Bispecific antibodies (BsAbs) have gained traction as an effective strategy for engaging macrophages in cancer immunotherapy by simultaneously targeting tumor antigens and macrophage-activating receptors. These antibodies are designed to bridge tumor cells and immune effectors, enhancing phagocytosis and promoting a pro-inflammatory TME. BsAbs targeting HER2, EGFR, and CD20 in combination with Fcγ receptors (FcγRI, FcγRIII) or CD40 have demonstrated preclinical efficacy in enhancing tumor clearance and activating both innate and adaptive immune responses (219).

Early clinical trials in the 2000s and 2008 explored BsAbs targeting HER2^+^ and EGFR^+^ tumors, such as MDX-210, MDX-H210, and MDX-447, which aimed to engage macrophages *via* Fcγ receptors. Despite being well tolerated, these early constructs failed to demonstrate significant anti-tumor efficacy, leading to their discontinuation (220). However, advancements in BsAb engineering and a deeper understanding of TAM biology have renewed interest in this approach, with newer-generation bispecifics now showing greater promise.

Recent clinical developments have introduced BsAbs with enhanced targeting precision and immune activation mechanisms. Ivonescimab (AK112), a PD-1/VEGF-A bispecific, is being evaluated in NSCLC and has been approved in China (221). Cadonilimab (AK104), a PD-1/CTLA-4 BsAb, has shown efficacy in relapsed/metastatic cervical cancer (222). In hematologic malignancies, TNB-486 (CD19/CD3) (223) and epcoritamab (CD20/CD3) (224) are demonstrating promising outcomes in B-cell lymphomas, while blinatumomab, an FDA-approved CD19/CD3 BsAb, remains a key treatment for acute lymphoblastic leukemia (225). These newer BsAbs not only employ macrophage activation but also integrate T cell recruitment, broadening their therapeutic impact and reaffirming their potential in next-generation cancer immunotherapy. However, clinical benefit as monotherapy has so far been limited, highlighting the need for rational combinations and biomarker-driven patient selection.

In conclusion, macrophage reprogramming strategies, CD47/SIRPα blockade, CD24/Siglec-10 blockade, TREM2 inhibition, and bispecific macrophage engagers, represent key avenues for restoring macrophage function and enhancing tumor clearance. While clinical trials for CD47 blockade are already yielding compelling results, newer targets like TREM2 and Siglec-10 are emerging as complementary strategies to further expand the therapeutic landscape of macrophage-based immunotherapy.

These new drug candidates illustrate the expansion of macrophage-targeted therapies beyond CSF-1R inhibitors, which have long been a foundational strategy in TAM modulation. CSF-1R inhibitors work by depleting immunosuppressive TAMs, aiming to shift the TME toward an anti-tumor state. However, despite robust macrophage depletion in preclinical models, clinical success has been limited, with most trials reporting low response rates as tumors adapt by recruiting alternative myeloid cells or activating compensatory immunosuppressive pathways (59). This shift includes the development of myeloid checkpoint inhibitors, such as anti-Siglec-15 and anti-TREM1, as well as agonistic therapies that actively stimulate macrophages toward a pro-inflammatory state. Siglec-15 is an immunosuppressive molecule expressed on TAMs, and its inhibition can enhance anti-tumor immunity (226). Similarly, targeting TREM1, an amplifier of inflammation, can modulate macrophage activity within the TME (227). Additionally, TLR4 agonists have been explored to activate macrophages, leading to the secretion of pro-inflammatory cytokines and chemokines, thereby promoting anti-tumor responses (228). CD40 agonists are being investigated to enhance macrophage activation and antigen presentation (229), while STING agonists aim to trigger innate immune sensing pathways within TAMs, further promoting T cell recruitment and tumor clearance (230).

Beyond macrophages, similar checkpoint and reprogramming strategies are also being investigated in other myeloid populations. For instance, CXCR2 antagonists such as SX-682 are being tested to block neutrophil recruitment and reprogram them away from the tumor-promoting N2 phenotype (NCT03161431) (231). Likewise, all-trans retinoic acid (ATRA) and phosphodiesterase-5 inhibitors have been evaluated to reduce the number and suppressive function of myeloid-derived suppressor cells (MDSCs) in patients with solid tumors (232). However, compared with these early-phase approaches, macrophages remain the most extensively characterized and clinically advanced myeloid subset, with multiple targeted agents already in Phase 1/2 development.

These advancements reflect the growing versatility of macrophage-targeted strategies, highlighting the need for continued research into combination therapies and biomarker-driven patient selection to fully harness the potential of macrophage reprogramming in cancer immunotherapy.

### 5.2 Reprogramming macrophages

Re-education of TAMs from M2 to M1 phenotypes aims to shift their role from tumor support to immune activation. M2 macrophages are induced by IL-4 and IL-13 and are characterized by their immunosuppressive and pro-tumor functions. By contrast, M1 macrophages, activated by IFN-γ, produce pro-inflammatory cytokines and ROS, leading to tumor cell destruction (233). Reprogramming macrophages can be achieved through targeted delivery of immunomodulatory agents.

#### 5.2.1 Nanoparticle-based macrophage reprogramming

Recent advancements in nanoparticle-based TAM modulation have provided highly specific strategies to reprogram macrophages from an immunosuppressive M2 phenotype into a pro-inflammatory M1 state, enhancing their tumoricidal capacity. Nanoparticle-based approaches offer precision targeting, allowing for localized macrophage reprogramming within the TME while minimizing systemic toxicity.

One notable approach involves manganese dioxide-conjugated nanoparticles, which reduce tumor hypoxia, a major factor driving TAM immunosuppression, while simultaneously promoting M1 polarization. In a breast cancer model, treatment with these nanoparticles increased tumor oxygenation and down-regulated HIF-1α, enhancing the efficacy of chemotherapy (234). Similarly, lipid nanocarriers encapsulating anti-IL-10 and anti-IL-10R siRNA have been designed to block IL-10 signaling, a key driver of macrophage-mediated immunosuppression. Preclinical studies in liver cancer demonstrated that silencing IL-10 signaling enhanced cytotoxic immune responses, restoring macrophage-driven anti-tumor activity (235).

Other reprogramming strategies use mannose-coated nanoparticles, which exploit the high expression of CD206 on M2-like TAMs to selectively deliver immunostimulatory agents. For instance, lignin nanoparticles loaded with the TLR7/8 agonist resiquimod (R848) have been designed to target CD206-expressing macrophages, effectively inducing M1 polarization and enhancing anti-tumor immunity (236). Additionally, di-mannose-modified polymers carrying mRNA encoding M1-polarization-associated transcription factors have been developed to target the CD206 receptor on macrophages, promoting M1 polarization and suppressing tumor growth in various models (237).

​β-glucan-based nanoparticles have emerged as a promising strategy to enhance anti-tumor immunity by stimulating macrophage phagocytosis and promoting the release of pro-inflammatory cytokines. These nanoparticles interact with pattern recognition receptors such as Dectin-1 on macrophages, leading to their activation and polarization toward the M1 phenotype. This reprogramming enhances the production of cytokines like TNF-α and IL-6, which are crucial for mounting effective anti-tumor responses (238).

While these strategies focus on reprogramming TAMs into a pro-inflammatory state, nanoparticles can also be used for targeted macrophage depletion, which is explored in Section 5.3.4. This complementary approach aims to eliminate highly suppressive macrophage populations, further reshaping the TME for effective immune responses.

#### 5.2.2 Epigenetic reprogramming of TAMs

Beyond cytokine signaling and nanoparticle-based interventions, recent studies suggest that epigenetic reprogramming can provide a longer-lasting shift in macrophage phenotype. Epigenetic modifiers, such as histone methyltransferases and DNA methylation enzymes, regulate TAM polarization and their ability to suppress immune responses.

One key target is enhancer of zeste homolog 2 (EZH2), a histone methyltransferase that promotes the M2-like phenotype by suppressing pro-inflammatory genes. Inhibiting EZH2 activity has been shown to restore M1 macrophage function, leading to enhanced anti-tumor immunity. In preclinical models of breast and lung cancer, EZH2 inhibitors improved responses to ICIs by reversing TAM-mediated immunosuppression (239).

Similarly, DNA methyltransferase (DNMT) inhibitors are being explored as macrophage reprogramming agents. DNA methylation regulates TAM polarization by silencing immune-stimulatory genes. DNMT inhibitors, such as 5-aza-2'-deoxycytidine (5-aza-dC), have been shown to reprogram TAMs from an immunosuppressive M2 state to a pro-inflammatory M1-like phenotype, restoring antigen presentation and cytokine production (240). Their role in macrophage modulation is further explored in Section 7.2.

​Metabolic–epigenetic crosstalk plays a critical role in sustaining macrophage polarization. For example, α-ketoglutarate (α-KG) promotes Jmjd3-dependent histone demethylation that drives M2 polarization, whereas accumulation of succinate stabilizes HIF-1α and favors pro-inflammatory M1 activation [255]. Similarly, acetyl-CoA availability can influence histone acetylation, reinforcing the transcriptional programs of polarized macrophages. These metabolic shifts are not merely biochemical phenomena but directly affect treatment outcomes: tumors enriched in α-KG-driven M2-like TAMs are more resistant to chemotherapy and immune checkpoint inhibitors, while succinate-associated M1-like TAMs correlate with enhanced T cell infiltration and improved response rates. Targeting these pathways - for instance, by modulating α-KG/succinate balance or blocking M2-favoring epigenetic modifiers - could therefore sustain M1 polarization and potentiate the efficacy of immunotherapy and other anticancer treatments (241).

While these approaches show promise, a deeper understanding of the molecular mechanisms linking epigenetics and macrophage plasticity is needed. Further insights into DNA methylation, histone modifications, and metabolic control will be explored in Section 7, where macrophage epigenetic reprogramming is discussed in greater detail.

### 5.3 Macrophage depletion

Macrophage depletion, also called macrophage ablation, is a strategy to eliminate TAMs that contribute to tumor progression. While older methods such as bisphosphonates and trabectedin have been used, newer approaches focus on selective and precise targeting of TAMs using advanced technologies.

#### 5.3.1 Selective depletion strategies: antibody-based approaches

Antibody-based therapies have become a powerful tool for selectively depleting TAMs while preserving normal immune function. Anti-CSF-1R antibodies, such as lacnotuzumab, block the CSF1/CSF-1R axis, leading to TAM depletion and enhanced T cell infiltration in tumors (242). While CSF-1R inhibitors have shown promise in altering the TME, their effects can be limited by compensatory recruitment of alternative myeloid populations, necessitating more targeted depletion strategies.

A complementary approach aims to restrict the immunosuppressive activity of TAMs by preventing monocyte recruitment to the TME. Anti-CCR2/CCL2 agents, such as carlumab and plozalizumab, were developed to block monocyte trafficking; however, early-phase trials did not demonstrate significant tumor responses (243). These disappointing results illustrate a recurring issue: therapies highly effective in animal models often fail to translate into durable human responses. More recently, newer combination approaches are being explored to enhance macrophage modulation. A notable trial combined APX005M, a CD40 agonist that activates macrophages, with cabiralizumab, a CSF-1R inhibitor that depletes M2 TAMs, demonstrating a synergistic effect by promoting macrophage repolarization (244).

A promising next-generation strategy involves targeting MARCO, a scavenger receptor highly expressed on tumor-promoting TAMs but absent on homeostatic macrophages. MARCO plays a key role in shaping an immunosuppressive TME by triggering MEK/ERK/p90RSK/CREB signaling, leading to IL-10 production, PD-L1 upregulation, and Treg expansion, which collectively inhibit cytotoxic CD8^+^ T cells and NK cells (245). Blocking MARCO has been shown to restore anti-tumor immunity by reducing IL-10 levels, downmodulating Tregs, and enhancing NK cell-mediated cytotoxicity through TNF-related apoptosis-inducing ligand (TRAIL) release. Macomics is actively developing anti-MARCO antibodies to selectively eliminate M2-like TAMs, potentially offering a more precise and effective alternative to CSF-1R inhibitors. Combining anti-MARCO therapy with PD-1/PD-L1 blockade is emerging as a prospective strategy to overcome resistance to T cell-directed immunotherapy, particularly in solid tumors such as melanoma (246, 247).

Combinations are indeed a major trend in macrophage-targeted therapies, particularly in efforts to convert immunologically “cold” tumors into “hot” ones. In a 2022 study, blocking CSF-1R to alter TAM composition in combination with anti-PD-L1 therapy resulted in enhanced T cell infiltration into tumors that were previously unresponsive to checkpoint blockade. This finding highlights the synergistic potential of targeting TAMs alongside T cell-directed immunotherapies, reinforcing the rationale for CSF-1R inhibitors as part of combination strategies in clinical trials (248).

Selective depletion of pro-tumoral CD163^+^ macrophages, which play a critical role in tumor immunosuppression, has also emerged as a promising immunotherapeutic strategy. Anti-CD163 antibodies, such as OR2805, selectively target this subset, sparing other myeloid populations and reducing off-target depletion effects observed with broader myeloid inhibitors (249). Studies have shown that high levels of CD163^+^ TAMs are generally associated with poor patient outcomes in solid tumors (250), underscoring the therapeutic potential of targeting this macrophage subset.

BsAbs targeting both CD47 and SIRPα have been developed to enhance macrophage phagocytosis of cancer cells while simultaneously reducing TAM-mediated immunosuppression. These therapies contribute to the depletion of immunosuppressive TAMs while strengthening the tumoricidal activity of remaining macrophages. However, this strategy has already been explored in detail in Section 5.1.5., where its mechanisms and clinical applications are thoroughly discussed.

#### 5.3.2 Engineered CAR-T cells for TAM depletion

A new preclinical breakthrough in macrophage depletion involves the use of engineered CAR-T cells, a form of adoptive cell therapy where T cells are genetically modified to express chimeric antigen receptors (CARs) that enable targeted tumor recognition and destruction. While CAR-T therapy has been widely studied in hematologic malignancies, recent advancements have expanded its application to target TAMs. More details on CAR technology and its broader applications will be explored in Section 8.

Traditionally designed to attack tumor cells, CAR-T therapies have now been reprogrammed to recognize macrophage-specific antigens within the TME. In mouse models of pancreatic, ovarian, and lung cancer, researchers developed CAR-T cells that specifically target macrophage surface markers, resulting in the efficient depletion of immunosuppressive TAMs, increased cytotoxic T cell infiltration, and enhanced tumor regression, ultimately reversing the immunosuppressive microenvironment. This approach not only shrank aggressive ovarian, lung, and pancreatic tumors in mice but also boosted overall anti-tumor immunity. The CAR-T cells not only cleared TAMs but also secreted IFN-γ, reactivating local immunity and amplifying the anti-tumor response. These findings pave the way for first-in-human trials of macrophage-targeting CAR-T therapies, offering a highly specific method for depleting pro-tumoral macrophages while preserving other myeloid cell populations (251).

#### 5.3.3 Macrophage depletion *via* metabolic interference

Another attractive strategy focuses on disrupting macrophage-specific metabolic pathways to selectively deplete TAMs. Unlike other immune cells, TAMs rely heavily on FAO and oxidative phosphorylation for survival, making them particularly susceptible to metabolic inhibitors (233).

Inhibitors of CPT1A, a key enzyme in FAO, have been shown to impair TAM survival while sparing other myeloid cells. Preclinical studies indicate that CPT1A blockade reduces tumor growth by selectively eliminating M2-like TAMs, which are highly dependent on FAO for energy (252).

Similarly, IACS-010759, an inhibitor of oxidative phosphorylation, has been found to selectively reduce TAM populations in the TME, leading to a pro-inflammatory shift and improved response to immunotherapy. Preclinical studies have demonstrated that combining IACS-010759 with radiotherapy or ICIs enhances therapeutic efficacy. For example, in a murine lung cancer model, the addition of IACS-010759 to radiotherapy and anti-PD-1 therapy not only prolonged survival but also induced systemic anti-tumor responses, including abscopal effects on unirradiated tumors (253).

By leveraging metabolic vulnerabilities unique to TAMs, these therapies offer a novel and selective method of macrophage depletion that minimizes the risk of systemic myeloid suppression.

#### 5.3.4 Nanoparticle-based targeted macrophage depletion

In addition to their role in macrophage reprogramming (discussed in Section 5.2.1.), nanoparticle-based strategies are also being developed to deplete immunosuppressive TAMs with high precision, reducing off-target effects associated with systemic depletion methods such as CSF-1R inhibitors.

One widely studied approach involves mannose-coated nanoparticles that engage CD206 expression on M2-like TAMs to deliver cytotoxic agents, such as doxorubicin, directly into immunosuppressive macrophages. This targeted delivery minimizes systemic toxicity while ensuring efficient TAM depletion within tumors (254).

A particularly promising strategy involves the use of pH-gated nanoparticles designed to regulate lysosomal function specifically in TAMs, leading to their selective depletion. By exploiting the acidic TME, these nanoparticles release their cytotoxic payload within TAMs, thereby reducing their population and alleviating immunosuppression (255).

Another strategy utilizes alginate-based hydrogels loaded with nanoparticles encapsulating pexidartinib, a CSF-1R inhibitor. This system enables sustained release of pexidartinib at the tumor site, effectively depleting TAMs and enhancing the efficacy of immune checkpoint blockade therapies (256).

Macrophage depletion remains a key strategy in modulating the TME and overcoming macrophage-driven immunosuppression. While traditional methods such as CSF-1R inhibition have shown some success, newer strategies, including MARCO-targeting antibodies, CD163 depletion, metabolic disruption, and CAR-T cells engineered to eliminate TAMs, are paving the way for more precise and effective macrophage depletion therapies. Nanoparticle-based depletion strategies are also gaining traction, providing highly targeted delivery of cytotoxic agents to TAMs while minimizing systemic toxicity.

With ongoing clinical trials and preclinical advancements, the next generation of macrophage depletion therapies holds great promise for improving response rates to immunotherapy and reducing tumor progression across multiple cancer types. Beyond depletion, other approaches such as RNA-based and epigenetic modulation aim to reprogram macrophages more durably, representing a natural continuation of the strategies discussed above.

## 6 Macrophage-targeted RNA-based therapies

RNA-based therapies are emerging as a powerful tool in immuno-oncology, offering precise control over gene expression in immune cells, including macrophages. Unlike traditional small-molecule drugs or protein-based therapeutics, RNA-based approaches provide the flexibility to silence pathogenic genes through siRNA and miRNA or to enhance beneficial immune functions using messenger RNA (mRNA), circular RNA (circRNA), and small activating RNA (saRNA). Given the central role of macrophages in the TME, these strategies aim to either inhibit tumor-promoting TAMs or enhance the anti-tumor activity of pro-inflammatory macrophages. Recent advancements in RNA delivery systems, such as lipid nanoparticles (LNPs), have significantly improved the stability and targeted uptake of RNA therapeutics, making macrophage-targeted RNA therapies a promising avenue for cancer treatment. Despite promising early-phase findings (e.g., MTL-CEBPA), challenges in efficient delivery and sustained activity have so far limited broad clinical success.

### 6.1 siRNA and miRNA therapies for TAM reprogramming

One of the most widely explored RNA-based approaches in macrophage modulation involves the use of siRNA and miRNA to selectively knock down immunosuppressive genes in TAMs. Tumors actively reprogram macrophages to adopt an anti-inflammatory, M2-like phenotype through signaling pathways mediated by CSF-1R, signal transducer and activator of transcription 3 (STAT3), and interferon regulatory factor 4 (IRF4). Silencing these pathways can reverse the immunosuppressive phenotype of TAMs, restoring their capacity to mount an effective anti-tumor response.

In preclinical studies, LNPs loaded with siRNAs targeting CSF-1R have been shown to deplete TAMs or reprogram them toward a pro-inflammatory phenotype. A pH-sensitive cationic lipid-based LNP effectively delivered siRNA to TAMs in a human tumor xenograft model, achieving efficient gene silencing, reducing tumor growth, and enhancing responses to ICIs (235). Similarly, inhibition of STAT3, a key transcription factor that promotes M2 polarization, has been demonstrated to restore M1-like characteristics in macrophages, increasing their ability to produce pro-inflammatory cytokines such as IL-12 and TNF-α (257).

Additionally, miRNAs such as miR-155 have been identified as critical regulators of macrophage polarization. By targeting suppressor of cytokine signaling 1 (SOCS1), miR-155 promotes M1 polarization and enhances pro-inflammatory cytokine production, while its silencing drives M2 polarization. Therapeutic approaches using miR-155 mimics have demonstrated potential in reprogramming TAMs toward a pro-inflammatory phenotype, thereby enhancing anti-tumor immunity (258).

### 6.2 mRNA-based activation of anti-tumor macrophages

Beyond gene silencing, mRNA-based therapies offer an alternative strategy to actively enhance the anti-tumor activity of macrophages by providing them with the genetic instructions to produce immunostimulatory proteins. A notable approach involves delivering *in vitro*-transcribed mRNA encoding M1-polarizing transcription factors directly into TAMs using targeted nanocarriers. Specifically, nanoparticles have been engineered to deliver mRNA encoding interferon regulatory factor 5 (IRF5) along with its activating kinase IKKβ. This strategy effectively reprograms TAMs from an immunosuppressive M2-like phenotype to a pro-inflammatory M1-like state, thereby promoting anti-tumor immunity and inducing tumor regression. Importantly, this method has demonstrated efficacy in various tumor models, including ovarian cancer, melanoma, and GBM, without causing systemic toxicity or disrupting immune homeostasis (237).

More recent studies have demonstrated that delivering mRNA encoding IFN-β directly into the TME can effectively stimulate anti-tumor immune responses. For instance, intratumoral administration of LNPs encapsulating IFN-β mRNA has been shown to inhibit tumor growth significantly. This approach enhances the activation of immune cells, including macrophages, leading to increased production of pro-inflammatory cytokines and improved recruitment of effector immune cells to the tumor site. Notably, these treatments have resulted in a higher ratio of CD8^+^ to CD4^+^ T cells and increased presence of M1-like macrophages within tumors, contributing to a more robust anti-tumor immune response (259).

These mRNA-based interventions leverage macrophages’ ability to efficiently take up and translate exogenous nucleic acids, making them promising candidates for *in situ* immune activation within the TME.

### 6.3 Small activating RNA for macrophage reprogramming

A novel class of RNA-based therapeutics, saRNA, is considered an alternative to siRNA-mediated gene silencing, offering a way to boost gene expression rather than inhibit it. One of the most notable examples is MTL-CEBPA, the first saRNA-based therapy to reach clinical trials. MTL-CEBPA activates CEBPA, a transcription factor that plays a critical role in myeloid differentiation and macrophage polarization (260).

In a Phase 1 trial for advanced liver cancer, MTL-CEBPA demonstrated promising anti-tumor effects by reprogramming TAMs from an immunosuppressive M2 phenotype to a pro-inflammatory M1 state. This shift in macrophage polarization restored T cell activation and improved response rates when combined with standard-of-care therapies. Notably, MTL-CEBPA combined with sorafenib induced tumor regression in 27% of patients, including a few complete responses, providing proof-of-concept for RNA-based macrophage epigenetic modulation in cancer therapy (260).

### 6.4 circRNA-based macrophage modulation

A novel and emerging RNA-based approach in macrophage modulation is the use of circRNA therapeutics. Unlike linear RNAs, circRNAs are highly stable due to their closed-loop structure, which makes them resistant to exonuclease degradation. This stability enables circRNAs to serve as more durable regulators of macrophage gene expression compared to traditional siRNA or mRNA approaches.

Recent studies have demonstrated that circRNAs can modulate macrophage polarization by acting as molecular sponges for miRNAs or directly interacting with transcription factors involved in immune regulation. By selectively expressing circRNAs that inhibit macrophage M2 polarization while promoting M1 activation, researchers aim to develop long-lasting interventions that sustain macrophage reprogramming within the TME (261).

Furthermore, engineered circRNAs encoding immunostimulatory proteins, such as IFN-γ or GM-CSF, offer a new dimension to macrophage-targeted therapies by combining the stability of circRNA with the functional benefits of mRNA-based immune activation (261). This emerging field represents a promising avenue for long-term macrophage reprogramming in cancer treatment.

RNA-based therapies provide a highly specific and adaptable strategy for modulating macrophage function in cancer and beyond. Clinical results published in 2021 validate the concept of reprogramming myeloid cells *via* RNA-based epigenetic modulation, demonstrating its potential in reshaping the TME (260). Building on this, researchers are now investigating additional epigenetic targets in TAMs that could further enhance their tumoricidal potential. Inhibiting key histone modifiers or targeting metabolic enzymes in macrophages has been shown to drive a sustained shift toward an M1-like phenotype, reinforcing the role of epigenetic modulation in immune activation. For instance, PI3Kγ inhibition in TAMs (using the drug eganelisib) has demonstrated enhanced anti-tumor immunity in preclinical models and is now undergoing early-phase clinical trials in combination with checkpoint inhibitors for solid tumors (262). Whether through siRNA- and miRNA-mediated gene silencing, mRNA-driven immune activation, saRNA-based gene upregulation, or the emerging field of circRNA-based reprogramming, these approaches offer exciting possibilities for harnessing macrophages as powerful immune mediators. With continued advancements in RNA delivery and stability, macrophage-targeted RNA therapeutics and epigenetic modulators are poised to become a transformative component of next-generation immunotherapy, offering durable immune reprogramming and improving responses to existing treatments. Building on this, epigenetic modifications such as histone regulation, DNA methylation, and chromatin remodeling provide an additional layer of durable control over macrophage phenotype, and are explored in the following section.

## 7 Macrophage epigenetic reprogramming

Epigenetic modifications represent a novel frontier in macrophage-targeted therapies, offering the potential to induce long-lasting functional changes without directly altering genetic sequences. Unlike conventional approaches that rely on depleting TAMs or transiently reprogramming them through cytokine signaling, epigenetic reprogramming provides a way to durably shift macrophage polarization and immune function. By modulating histone modifications, DNA methylation, and chromatin accessibility, researchers aim to override tumor-induced immunosuppression and sustain macrophages in a pro-inflammatory, anti-tumor state. The ability to epigenetically rewire macrophages has profound implications not only for cancer immunotherapy but also for chronic inflammatory diseases and trained immunity.

### 7.1 Histone modifications and TAM reprogramming

One of the primary mechanisms by which macrophage function is epigenetically regulated is through histone modifications, which control the accessibility of transcriptional machinery to key immune genes. Histone methylation and acetylation serve as epigenetic switches that determine whether macrophages adopt a tumor-promoting or tumoricidal phenotype (263).

The enzyme EZH2, a key histone methyltransferase, contributes to the immunosuppressive state of TAMs by mediating H3K27me3 deposition, which represses genes involved in macrophage activation. Rather than directly suppressing TNF-α and IL-12, EZH2 inhibition has been shown to alter macrophage polarization by modulating metabolic and inflammatory pathways. Studies indicate that pharmacological inhibition of EZH2 can reduce M2-like characteristics in TAMs, leading to a shift toward a more pro-inflammatory phenotype that enhances anti-tumor immune responses (264). Recent studies have also demonstrated that pharmacological inhibition of EZH2 not only reactivates pro-inflammatory macrophage functions but also enhances response to immune checkpoint blockade therapy, relieving the immunosuppressive influence of TAMs within the TME (265).

Another promising target is KDM6B (JMJD3), a histone demethylase that acts as a counterbalance to EZH2 by removing H3K27me3 repressive marks. KDM6B activation has been shown to promote pro-inflammatory macrophage phenotypes, enhancing their ability to produce IL-12 and present antigens to T cells. Preclinical models suggest that KDM6B activation synergizes with checkpoint inhibitors, further improving anti-tumor immunity (266). These findings highlight the potential for histone modification inhibitors as a means to epigenetically shift TAMs toward an anti-tumor state.

### 7.2 DNA methylation and macrophage activation

DNA methylation is a key epigenetic mechanism regulating macrophage function, primarily through the addition of methyl groups to cytosine residues in CpG islands, leading to the silencing of immune-stimulatory genes. In TAMs, aberrant DNA methylation reinforces an immunosuppressive phenotype by downregulating genes critical for antigen presentation, inflammatory cytokine production, and phagocytosis. These modifications contribute to the maintenance of an M2-like state, limiting the ability of macrophages to mount an effective anti-tumor response (240, 266).

Targeting DNMTs with small-molecule inhibitors is increasingly recognized as a viable means of restoring anti-tumor macrophage function. Preclinical studies have shown that treatment with DNMT inhibitors, such as 5-aza-dC, can reprogram TAMs by increasing the expression of MHC class II molecules and pro-inflammatory cytokines like IL-12 and TNF-α, thereby enhancing antigen presentation and promoting a stronger adaptive immune response. Furthermore, a combination approach using 5-aza-dC alongside the histone deacetylase (HDAC) inhibitor trichostatin A has demonstrated the ability to shift M2 macrophages toward an M1-like phenotype. This epigenetic therapy not only altered cytokine secretion patterns, reducing M2-associated cytokines while increasing M1 markers, but also sensitized tumor cells to paclitaxel, leading to improved anti-tumor immunity in murine models (240).

### 7.3 Chromatin remodeling and trained immunity-based macrophage reprogramming

A growing body of research focuses on chromatin remodeling as a means of inducing trained immunity in macrophages, enabling them to mount enhanced responses upon secondary stimulation. Unlike classical immune memory mediated by adaptive immune cells, trained immunity relies on epigenetic and metabolic modifications that prime macrophages for heightened inflammatory activity. Chromatin accessibility, dynamically regulated by chromatin-modifying complexes such as the SWI/SNF family, plays a central role in this process by dictating whether transcription factors can access pro-inflammatory gene *loci* (267). Researchers aim to harness these mechanisms to develop macrophage-targeted therapies that sustain anti-tumor immunity, reinforcing long-term resistance against tumor-induced immunosuppression.

Trained immunity-based macrophage reprogramming relies on epigenetic modifications that enhance long-term macrophage responsiveness to inflammatory stimuli. β-glucans, discussed in Section 5.2.1., are one of the key inducers of this phenomenon, promoting histone acetylation and chromatin remodeling to sustain a pro-inflammatory macrophage phenotype.

One of the most well-characterized inducers of trained immunity with clinical relevance is Bacillus-Calmette-Guérin vaccination, primarily known for its role in bladder cancer immunotherapy. This vaccination has been shown to epigenetically reprogram macrophages, enhancing H3K4 trimethylation at promoters of pro-inflammatory genes, thereby boosting cytokine production such as TNF-α and IL-6 (268). This non-specific immune-enhancing property is currently being explored for applications beyond bladder cancer, particularly in settings where macrophage activation can improve anti-tumor responses.

Recent research has identified novel metabolic and epigenetic interventions as promising strategies for inducing durable macrophage activation. The mammalian target of rapamycin complex 1 (mTORC1) pathway has been implicated in sustaining long-term macrophage activation in cancer models. Pharmacological modulation of mTORC1 activity enhances glycolysis and histone modifications, reinforcing a persistent anti-tumor phenotype in macrophages. Studies suggest that targeting mTORC1 in TAMs may enhance macrophage resistance to tumor-derived immunosuppressive signals and improve responses to ICIs (269).

HDAC inhibitors are being explored for their ability to lock macrophages into a pro-inflammatory trained immunity state. By maintaining histone acetylation at key *loci*, HDAC inhibitors prevent macrophages from reverting to an immunosuppressive M2-like phenotype. Preclinical studies indicate that HDAC inhibition enhances the persistence of tumoricidal macrophages, making them more effective partners for ICIs and adoptive cell therapies (270, 271).

Sirtuins (SIRT1, SIRT3, SIRT6), a family of NAD^+^-dependent deacetylases, have emerged as potential targets for epigenetic priming of macrophages in cancer therapy. Studies suggest that modulating sirtuin activity can enhance macrophage inflammatory responses and antigen presentation, making TAMs more resistant to tumor-driven reprogramming. Sirtuin inhibitors or activators are currently under investigation to assess their ability to sustain anti-tumor macrophage activity *in vivo* (271).

Epigenetic reprogramming of macrophages represents a groundbreaking approach to immunotherapy, providing a means to durably alter macrophage function without direct genetic modifications. By targeting histone modifications, DNA methylation, and chromatin accessibility, researchers aim to rewire TAMs toward a sustained pro-inflammatory phenotype, enhancing anti-tumor immunity.

The inhibition of EZH2 and activation of KDM6B have demonstrated strong potential in preclinical models, while DNMT inhibitors offer another route to restoring pro-inflammatory macrophage functions. Trained immunity-based approaches using β-glucans are emerging as a promising way to sustain long-term macrophage activation, preventing TAMs from reverting to an immunosuppressive phenotype.

Although challenges related to specificity and delivery remain, ongoing advances in nanotechnology and epigenomic profiling hold promise for translating macrophage epigenetic therapies into clinical applications. With continued research, epigenetic reprogramming strategies could become a key component of next-generation cancer immunotherapy, offering new hope for durable immune activation in tumors.

In parallel to pharmacological and molecular reprogramming, cell-based approaches such as CAR-macrophages and engineered macrophages provide a complementary strategy, directly harnessing macrophages themselves as living therapeutics.

## 8 Macrophage-based adoptive cell therapies

Adoptive cell therapy has emerged as a promising strategy in cancer immunotherapy, leveraging the innate plasticity and tumor-infiltrating capacity of immune cells to enhance anti-tumor responses. While T cell-based therapies, such as CAR-T cells, have demonstrated remarkable efficacy in hematologic malignancies, their success in solid tumors remains limited due to the immunosuppressive TME and physical barriers preventing efficient infiltration. Macrophage-based adoptive cell therapies offer an alternative approach, exploiting the ability of macrophages to infiltrate solid tumors, modulate immune responses, and deliver therapeutic agents. These strategies aim to reprogram TAMs toward a pro-inflammatory phenotype, use macrophages as carriers for targeted drug delivery, or harness EVs to modulate the immune landscape of the TME. While preclinical results are compelling, no clinical data yet validate these platforms, and successful translation will require overcoming hurdles related to scalability, persistence, and safety.

### 8.1 CAR-macrophages: a new frontier in adoptive therapy

An important early clinical attempt in macrophage-based cell therapy is chimeric antigen receptor macrophages (CAR-M), which are genetically engineered to express tumor-specific CARs, enabling them to selectively target and phagocytose cancer cells. Unlike CAR T cells, which rely on direct cytotoxicity, CAR-Ms offer the additional advantage of antigen presentation, stimulating an adaptive immune response. Moreover, CAR-M therapy does not rely on MHC compatibility, making it a broadly applicable approach across different tumor types (272).

An early but noteworthy step in CAR-M development was the first-in-human Phase 1 trial of CT-0508, a CAR macrophage targeting HER2^+^ solid tumors (NCT04660929). This study demonstrated that CT-0508 therapy was safe, with no dose-limiting toxicities observed. Although no complete responses were noted, stable disease was achieved in 28.6% of patients (4 of 14) whose tumors showed high HER2 expression (HER2 IHC score of 3+). Biopsies revealed increased T cell infiltration and activation of the TME, suggesting that CAR-M therapy may function synergistically with T cell-based immunotherapies (196). Although safe and biologically active, CAR-M therapy has not yet produced objective responses, emphasizing the need for further optimization before clinical impact can be realized.

Following the discontinuation of CT-0508, Carisma Therapeutics has shifted its focus to CT-0525, an *ex vivo* gene-modified autologous CAR-monocyte therapy designed for HER2^+^ solid tumors. The ongoing Phase 1 trial (NCT06254807) aims to evaluate the safety, tolerability, and feasibility of CT-0525 manufacturing, with completion expected in March 2026 (273). This next-generation CAR-M approach builds on the knowledge gained from CT-0508, with the goal of improving therapeutic efficacy and persistence within the TME.

### 8.2 Next-generation CAR-M approaches

To improve efficacy and manufacturing efficiency, several next-generation CAR-M approaches are in development.

One such approach is mRNA-based CAR-M (MCY-M11), developed by Carisma Therapeutics. This macrophage therapy utilizes mRNA electroporation to introduce CAR constructs into monocytes, eliminating the need for viral vectors. A Phase 1 trial is currently evaluating MCY-M11 in ovarian cancer and peritoneal mesothelioma, with preliminary data indicating increased macrophage tumor infiltration (274).

Another promising development is iPSC-derived CAR-M (SY001), representing the first off-the-shelf CAR-M product. This induced pluripotent stem cell (iPSC)-derived macrophage reduces patient-specific variability and enhances scalability. SY001 is currently being tested in preclinical models of solid tumors, offering a potential solution for mass production of CAR-M therapies (275).

Additionally, CD5 CAR-M (MT-101) has been developed by Myeloid Therapeutics as an mRNA-engineered CAR-M targeting CD5. This therapy is currently undergoing evaluation in a Phase 1/2 trial for T cell lymphoma (NCT05138458). The study aims to determine whether CAR-M therapies can be effectively applied beyond solid tumors, expanding their potential clinical applications (276).

### 8.3 Engineered macrophages for immunotherapy

Beyond CAR-macrophages, engineered macrophages have been designed to release therapeutic molecules directly within the TME, amplifying anti-tumor immunity. One notable example involves macrophages engineered to secrete IL-12, a cytokine that enhances cytotoxic T cell activity and promotes a pro-inflammatory TME. Preclinical studies in GBM and pancreatic cancer demonstrated increased tumor regression following the infusion of IL-12-secreting macrophages, and clinical translation is currently underway (277, 278).

Similarly, TRAIL-expressing macrophages represent a targeted approach to tumor eradication. These macrophages are engineered to express TRAIL, a protein that selectively induces apoptosis in tumor cells expressing TRAIL receptors, specifically within the TME. Studies in breast and colon cancer models have shown that TRAIL-expressing macrophages infiltrate tumors and, particularly when expressing a trimeric form of TRAIL (Tri-TRAIL) compared to a monomeric form (Mono-TRAIL), trigger cancer cell death, reducing tumor burden while sparing normal tissues (279).

A novel approach involves engineering macrophages to act as inflammation-triggering entities, thereby enhancing the efficacy of ICIs. A study reported the development of "MacTriggers," engineered macrophages designed to induce an inflammatory environment specifically within tumor tissues. Upon intravenous administration in mouse models, these MacTriggers upregulated the expression levels of immune checkpoint proteins, including PD-1 on CD8^+^ T cells and PD-L1 on cancer cells and macrophages. This upregulation led to a significant enhancement of the anti-tumor effects of ICIs, suggesting that engineered macrophages can effectively modulate the TME to improve responses to checkpoint blockade therapies (280).

### 8.4 Macrophage-derived EVs as a therapeutic platform

In contrast to tumor-derived vesicles described in Section 2, which promote monocyte recruitment and immunosuppression, macrophage-derived EVs are now being engineered as therapeutic tools. These EVs can be loaded with proteins, siRNA, miRNA, or mRNA, enabling modulation of immune and tumor cells within the TME. Their inherent biocompatibility and tropism for tumor tissues make them attractive for targeted delivery. Preclinical studies show that M1-EVs loaded with paclitaxel reduced breast tumor volume by ~45 % over two weeks compared to controls (268), and EVs bearing si CX3CR1 in PDAC models achieved ~50 % knockdown in TAMs and ~30 % slower tumor growth (281). These results highlight macrophage-derived EVs as a versatile and promising cell-free immunotherapy platform.

EVs offer several advantages over traditional cell-based therapies. They can cross biological barriers, making them particularly effective in brain tumors and metastatic cancers. Additionally, EVs exhibit minimal immunogenicity, reducing the risk of rejection and allowing for safer administration. Their stability in circulation ensures efficient delivery of therapeutic payloads to the tumor site (282).

These unique properties position macrophage-derived EVs as a potentially useful cell-free alternative, though their utility remains to be clinically confirmed.

### 8.5 Macrophages as vehicles for drug delivery

Genetically modified macrophages have been extensively explored as drug delivery vehicles, utilizing their natural ability to home to tumors and integrate into the TME. Researchers are now developing innovative ways to exploit macrophages’ tumor-homing properties to enhance therapeutic delivery. One emerging strategy is using macrophages as “Trojan horses” to transport and release therapeutic agents directly within the TME. Unlike synthetic nanoparticles or free drug formulations, macrophages naturally infiltrate tumors, making them an ideal vehicle for targeted therapy.

One promising approach involves loading monocytes with oncolytic viruses to enhance virotherapy. In preclinical models, monocytes have been engineered to carry oncolytic viruses into tumors, where the virus selectively infects and kills cancer cells. This macrophage-mediated delivery strategy overcomes traditional barriers to virotherapy, including immune clearance and poor penetration into solid tumors. Studies in 2023 are testing this concept for hard-to-reach malignancies such as gliomas, where macrophages can effectively navigate the dense tumor architecture and deposit viral payloads in hypoxic regions (283).

### 8.6 Macrophage-drug conjugates: a novel delivery strategy

A novel experimental platform in this field is the introduction of macrophage-drug conjugates (MDCs), a novel technology designed to enhance the therapeutic potential of macrophage-based therapies. Developed by Cellis, MDCs capitalize on the innate tumor-homing properties of macrophages while providing a versatile platform for drug delivery. These conjugates can be loaded with a wide range of therapeutic agents, including chemotherapeutics, RNA-based drugs, and immunomodulatory molecules, allowing for targeted and controlled drug release within the TME (284).

MDCs directly transfer drugs to cancer cells, improving the efficacy of traditional therapies while promoting long-term immune reprogramming. Preliminary studies suggest that MDCs achieve deep tumor penetration, making them highly effective in dense, fibrotic tumors. They have demonstrated improved anti-tumor efficacy, leading to prolonged survival in preclinical models. Additionally, MDCs induce durable immune memory, reducing the likelihood of tumor recurrence (285).

By integrating MDCs with other macrophage-based immunotherapies, researchers aim to create a multi-functional platform that not only targets tumors but also reshapes the immune microenvironment for sustained therapeutic benefit (285, 286). The ability of MDCs to deliver drugs directly to the tumor site represents a significant advancement in macrophage-based cell therapy, showing promise in preclinical models, though clinical translation remains to be established.

Recent work described an MDC platform employing ferritin–drug conjugates (FT-735) that enables macrophages to transfer payloads to cancer cells through a contact-dependent mechanism termed TRAIN (TRAnsfer of Iron-binding protein) (206). In murine models of colorectal and breast cancer, MDC administration reduced tumor volume by 40–60% compared with unconjugated controls and extended median survival by approximately 35% (285).

It was further demonstrated that in orthotopic glioblastoma models, MDCs loaded with monomethyl auristatin E (MMAE) achieved complete tumor regression in 3 of 10 treated mice and significantly prolonged survival (median 48 vs. 28 days in controls) (206, 285). These effects were associated with reprogramming of the TME, including a two-fold increase in CD8^+^ T-cell infiltration and a 50% reduction in regulatory T-cell frequency. Importantly, rechallenge experiments showed protection in 70% of surviving animals, indicating durable immune memory (285).

Translational studies using dissociated patient glioblastoma samples confirmed efficient transfer of ferritin–drug payloads from MDCs to tumor cells *ex vivo*, supporting the feasibility of the platform under human TME conditions (285).

Despite these promising data, all findings remain preclinical. Challenges include tumor heterogeneity, which influences transfer efficiency, and the need for large-scale standardization of ferritin–drug conjugate loading (285). No clinical trial of MDCs has yet been reported.

Macrophage-based adoptive cell therapies are rapidly evolving, with CAR-macrophages, engineered macrophages, EVs, and MDCs representing diverse and complementary strategies. The feasibility and safety of CT-0508 have been demonstrated, though efficacy remains modest and further optimization is required, and next-generation approaches such as iPSC-derived and mRNA-based CAR-Ms are further improving their scalability and efficacy. Meanwhile, engineered macrophages that release IL-12 or TRAIL, along with macrophage-derived EVs, provide alternative methods for reshaping the TME.

With continued preclinical advancements and clinical trials, macrophage-based therapies are poised to become a key component of the next generation of cancer immunotherapy, offering new hope for solid tumor treatment.

## 9 Challenges and future directions

Despite the excitement around macrophage-targeted therapies, several notable challenges remain that must be addressed to fully realize their clinical potential.

One of the major hurdles is TAM plasticity, which complicates therapeutic targeting: even if macrophages are successfully reprogrammed toward an M1-like state, tumor-derived signals can rapidly drive them back to an immunosuppressive phenotype (287). Moreover, common markers like CD206 and CD163, widely used to identify pro-tumoral TAMs, are also expressed on certain normal macrophages, such as CD206 on liver sinusoidal macrophages (288), raising concerns about off-target effects and systemic toxicity. Indeed, clinical experience with CSF-1R inhibitors (e.g., emactuzumab, pexidartinib) has shown class-specific toxicities such as hepatotoxicity and periorbital edema, while CD47 blockade has been associated with on-target anemia due to phagocytosis of red blood cells. These examples illustrate the need to refine TAM-selective biomarkers to minimize collateral toxicity. Future research should prioritize single-cell multi-omics (scRNA-seq, CyTOF, spatial transcriptomics) to define tumor-specific TAM subsets and generate next-generation biomarkers that enable more precise and selective interventions.

Biomarker selection is increasingly critical for translating TAM-targeted therapies into the clinic. For instance, high baseline expression of CD47 correlates with clinical benefit in patients receiving CD47–SIRPα blockade (e.g., magrolimab) (289), whereas M-CSF levels and CSF1R expression are being explored as predictors of response to CSF1R inhibitors such as emactuzumab (248). In CAR-M trials, tumor HER2 status (IHC 3+) was required for enrollment in the CT-0508 study, and responses were observed almost exclusively in patients with high HER2 expression (196). Similarly, exploratory analyses suggest that transcriptional TAM signatures enriched for M2 polarization (e.g., CD163, MSR1, MRC1) may predict resistance to checkpoint inhibitors, supporting the rationale for TAM reprogramming strategies.

Another major obstacle is the limited efficacy of TAM-targeted monotherapies. While TAM depletion and reprogramming strategies have demonstrated promising preclinical results, many early-phase clinical trials of CSF-1R inhibitors and CCR2/CCL2 inhibitors have failed to induce significant tumor regressions when used alone (290). Tumors can adapt by recruiting alternative suppressive myeloid cells or upregulating compensatory pathways, such as CXCLs and CCLs, to restore their immunosuppressive microenvironment. As a result, combination therapies are emerging as a critical strategy to enhance macrophage-targeted approaches. For instance, combining TAM-targeted agents with T cell checkpoint inhibitors (e.g., anti-PD-L1) has been shown to increase CD8^+^ T cell infiltration into previously unresponsive tumors. Early trials, such as CSF-1R blockade (emactuzumab) combined with PD-L1 inhibitors (atezolizumab), have provided proof-of-concept for synergistic approaches (291).Combination strategies must also consider overlapping toxicities - for example, increased immune-related adverse events when macrophage-modulating agents are combined with checkpoint inhibitors - highlighting the importance of carefully designed dosing and sequencing in clinical trials. Ongoing objectives include rational design of combination regimens, systematic biomarker discovery for patient stratification, and adaptive trial designs that can identify synergy and avoid redundant combinations.

In the field of macrophage-based adoptive cell therapies, engineering, persistence, and delivery remain technical barriers. CAR-M are significantly more difficult to modify genetically than T cells due to their innate nucleic acid-sensing pathways, which trigger antiviral defenses and reduce gene transfer efficiency. Future advancements must improve non-viral gene delivery methods (e.g., mRNA electroporation, transposon systems), optimize *in vitro* expansion protocols, and expand CAR-M cells efficiently while maintaining their functional stability *in vivo* (273). Additionally, combining CAR-M with checkpoint blockade, chemotherapy, or radiation therapy may further enhance tumor elimination by addressing multiple aspects of tumor progression simultaneously (292).

Beyond cell-based therapies, EVs derived from macrophages offer a cell-free approach to tumor targeting. Macrophage-derived EVs can efficiently carry immunomodulatory molecules, RNA therapeutics, and metabolic inhibitors into the TME, providing a versatile platform for TAM modulation. Key challenges include improving stability, enhancing specificity, and ensuring large-scale production. Engineering EVs with precise cargo loading and surface modifications could help optimize targeting of specific TAM subsets while avoiding systemic clearance (282). Further priorities include standardized GMP-compliant EV production, surface engineering with TAM-specific ligands, and development of scalable quality-control assays for EV cargo consistency.

RNA-based therapies are also gaining attention as a means for macrophage modulation, but challenges remain in delivery, selectivity, and stability (235, 259). Ensuring efficient uptake of RNA therapeutics by macrophages without affecting other immune cells is crucial. Research priorities should include the development of macrophage-specific targeting ligands, optimized lipid nanoparticle formulations with controlled release, and systematic preclinical evaluation of off-target immune activation to ensure safety before broader translation. Potential safety issues include innate immune activation (e.g., TLR sensing of exogenous RNA) and cytokine release, which must be addressed by optimizing chemical modifications and delivery formulations. Additionally, improving intracellular stability could boost therapeutic efficacy, and expanding macrophage-targeted RNA therapies beyond oncology to chronic inflammatory diseases, fibrosis, and autoimmune disorders may further increase their clinical impact.

Macrophage epigenetic reprogramming represents an exciting frontier, but significant hurdles remain. One major concern is specificity since global inhibition of histone modifiers or DNMTs could lead to unintended effects on other immune and non-immune cells. Developing macrophage-specific delivery systems for epigenetic modifiers is crucial to reduce off-target toxicity. Nanoparticle-based delivery of histone modification inhibitors or DNMT-targeting RNA molecules represents one promising strategy. Furthermore, the metabolic-epigenetic interplay in TAM polarization remains poorly understood (266). Future studies should dissect the role of metabolic intermediates (α-KG, acetyl-CoA, lactate) in shaping epigenetic marks, and design macrophage-targeted formulations of HDAC and DNMT inhibitors to minimize systemic toxicity.

An additional promising avenue is trained immunity, where macrophages undergo long-lasting epigenetic modifications, enabling them to mount stronger immune responses upon re-exposure to tumor antigens (268). β-glucans and other metabolic stimuli have been shown to induce a tumoricidal phenotype in macrophages, making them more resistant to tumor-derived immunosuppressive signals (238). Future objectives include evaluating trained immunity in oncology clinical trials, establishing safe dosing strategies, and testing its efficacy in combination with ICIs or CAR-M therapies to achieve durable responses (268).

From a technological perspective, improvements in delivery and manufacturing will be critical. For cell therapies (CAR-M, MDCs, etc.), new gene-editing methods and mRNA-based approaches may enhance efficiency and function while overcoming current challenges related to immune sensing and persistence (259). For drug delivery, approaches such as targeted nanoparticles and local administration (e.g., intratumoral injections, hydrogel depots) could reduce systemic toxicity while maximizing drug delivery to TAMs. *In situ* macrophage reprogramming using injectable cytokine gene therapies or small molecules is another promising area, with preclinical models demonstrating that CSF-1R inhibitors combined with TLR agonists can simultaneously deplete immunosuppressive TAMs and activate anti-tumor macrophages (59, 228). Such strategies could be further optimized for clinical translation. Next steps include development of intratumoral or hydrogel-based delivery platforms for localized TAM reprogramming, and modular GMP manufacturing pipelines that allow rapid adaptation of engineered macrophage products.

Despite these challenges, macrophage-targeted therapies continue to evolve, fueled by advancements in genetic engineering, RNA therapeutics, EVs, and epigenetic modulation. The integration of macrophage-directed approaches with next-generation combination therapies, including oncolytic viruses, metabolic modulators, and ICIs, holds great promise for overcoming resistance mechanisms and improving patient outcomes. As our understanding of macrophage biology deepens, new strategies will emerge to enhance specificity, durability, and therapeutic efficacy, paving the way for more effective treatments against solid tumors and other malignancies.

## 10 Conclusions

Innovative macrophage-targeted therapies are rapidly gaining traction in cancer immunotherapy, utilizing the ability of these cells to penetrate tumors, regulate immune responses, and directly destroy cancer cells. Advances in macrophage engineering, RNA-based therapies, epigenetic reprogramming, and nanoparticle-based delivery systems have significantly expanded the therapeutic potential of macrophage-directed strategies.

Between 2023 and 2025, macrophage-targeted therapies have progressed into Phase 1/2 trials across multiple cancer types, including solid tumors (breast, gastric, ovarian, lung) and hematologic malignancies. These investigational treatments range from engineered cell therapies to monoclonal antibodies and small-molecule inhibitors, with early outcomes demonstrating tolerable safety and signs of immune activation, such as increased T cell infiltration and stable disease. However, objective tumor regressions remain infrequent, underscoring the need for further trial optimization, biomarker-driven patient selection, and combination therapy strategies to enhance efficacy. Although macrophage-targeted therapies have generated substantial enthusiasm, clinical efficacy has often lagged behind preclinical expectations. Addressing tumor heterogeneity, compensatory immunosuppressive pathways, and safety concerns will be essential for successful translation.

While challenges such as macrophage plasticity, off-target effects, and delivery efficiency remain, innovative solutions, including improved engineering techniques, combination therapies, and macrophage-specific targeting, are rapidly advancing the field. The successful translation of macrophage-based therapies into clinical applications will depend on continued research into macrophage biology, improved delivery platforms, and strategic integration with existing immunotherapies.

With ongoing clinical trials and technological advancements, macrophage-directed therapies show promise in early clinical studies, but objective tumor regressions remain infrequent and substantial hurdles must be overcome to achieve durable benefit. It is important to recognize that macrophages are part of a broader myeloid landscape that includes neutrophils and MDSCs, both of which are also being explored as therapeutic targets. While these fields are still in earlier stages of development, the clinical maturity and functional versatility of macrophage-directed strategies currently place them at the forefront of myeloid immunotherapy. As our understanding of macrophage function deepens, these strategies may become a cornerstone of next-generation cancer immunotherapy, offering new hope for patients with solid tumors and other malignancies.

## Appendix I

### Table 1. Macrophage-targeting agents in clinical development/early approval.

**Table:**

Modality	Agent (target) / company	Indication(s)	Phase / trial ID	Delivery modality / route	Regimen (combo)	Key outcomes (latest)	Status
CD47/SIRPα blockade	Evorpacept (SIRPα-Fc) / ALX Oncology	HER2+ gastric/GEJ^1^ (2L/3L)	Ph2 randomized, ASPEN-06, NCT05002127	Systemic IV infusion	+ trastuzumab + ramucirumab + paclitaxel	ORR 48.9–52% vs 22% control; durable responses in updated 2024/2025 cuts	Ongoing; positive Ph2; biomarker work; registrational path TBD
CD47/SIRPα blockade	Evorpacept (SIRPα-Fc) / ALX Oncology	HNSCC (1L/2L)	Ph2 randomized, ASPEN-03/04 (HNSCC), e.g., NCT04675294	Systemic IV infusion	+ pembrolizumab (± chemo)	Did not meet primary endpoints	Programs not advancing in HNSCC
CD47 (mAb)	Magrolimab / Gilead	HR-MDS; AML (unfit; TP53mut)	Ph3 ENHANCE / ENHANCE-2 / ENHANCE-3	Systemic IV infusion	+ azacitidine (± venetoclax)	Futility and increased deaths; FDA full/partial clinical holds	Terminated / paused in MDS/AML and solid tumors
SIRPα-Fc (IgG1)	TTI-621 / Pfizer (Trillium)	Hematologic malignancies	Early phase (multiple)	Systemic IV infusion	± rituximab / chemo	Early signals reported historically; ongoing updates	Ongoing/early
SIRPα-Fc (IgG4)	TTI-622 / Pfizer (Trillium)	Hematologic malignancies	Early phase (multiple)	Systemic IV infusion	± rituximab	Early signals reported historically; ongoing updates	Ongoing/early
CSF1R inhibitor (SM)	Vimseltinib (Romvimza) / Deciphera	TGCT (diffuse/locally advanced, not resectable)	Ph3 MOTION - FDA-approved (14 Feb 2025)	Oral small-molecule, systemic	Monotherapy	Met ORR primary endpoint; QoL^2^ benefits	Approved (US)
CSF1R mAb	Emactuzumab (RG7155) / SynOx	TGCT (not amenable to surgery)	Ph3 TANGENT	Systemic IV infusion	Monotherapy	Prior dTGCT^3^ responses; favorable tolerability	Ph3 ongoing (enrollment completed; topline early 2026)
CSF1R mAb	Cabiralizumab (BMS-986227) / BMS	Pancreatic cancer	Ph2 randomized	Systemic IV infusion	+ nivolumab ± chemo vs chemo	No survival benefit; study ended	Negative
PI3Kγ inhibitor	Eganelisib (IPI-549) / Infinity	Urothelial carcinoma; TNBC^4^; melanoma (combos)	Ph2 MARIO-275 etc.	Oral small-molecule, systemic	+ nivolumab (± others)	Earlier ORR/OS^5^ deltas reported; sponsor entered bankruptcy; asset sale 2023–2024	Development uncertain
CD40 agonist mAb	Sotigalimab (APX005M) / Apexigen	mPDAC^6^ (1L)	Ph2 PRINCE randomized	Systemic IV infusion	+ gemcitabine / nab-paclitaxel (± nivolumab)	No OS improvement vs chemo	Negative
CD40 agonist mAb	SEA-CD40 / Seagen	Solid tumors; mPDAC	Ph1/2	Systemic IV infusion	+ gemcitabine / nab-paclitaxel + pembrolizumab	cORR^7^ ~44%, mPFS^8^ 7.4 mo, mOS^9^ 15.0 mo in Ph1 cohort; tolerable	Ongoing basket/combos
LILRB2/ILT4 (myeloid checkpoint)	JTX-8064 / Jounce	PROC^10^; solid tumors	Ph1/2 INNATE, NCT04669899	Systemic IV infusion	+ PD-1 (pimivalimab)	PROC cohort: deep/durable responses in preplanned review	Ongoing/strategic changes
Dual ILT2/ILT4	NGM707 / NGM Bio	Advanced solid tumors	Ph1/2, NCT04913337	Systemic IV infusion	± pembrolizumab	PK/RO^11^ achieved; on-treatment biomarker modulation	Ongoing
TREM2 (depleting TAMs)	PY314 / Pionyr	Solid tumors; PROC cohort explored	Ph1a/1b, NCT04691375	Systemic IV infusion	± pembrolizumab	Acceptable safety (low ≥G3 TRAEs ^12^); rationale for further study	Ongoing
CAR-macrophage	CT-0508 (HER2 CAR-M) / Carisma	HER2+ solid tumors	Ph1, NCT04660929	*Ex vivo* modified macrophages; reinfused IV	Autologous CAR-M (± preconditioning)	Interim: feasible manufacturing, safety; signals of activity	Ongoing
CAR- monocyte	CT-0525 (HER2 CAR-monocyte) / Carisma	HER2+ solid tumors	Ph1, NCT06254807 (Fast Track)	*Ex vivo* modified macrophages; reinfused IV	Autologous CAR-monocytes	First-in-human; dosing initiated 2024	Ongoing
CAR- macrophage	MCY-M11 (mesothelin CAR-M) / MaxCyte	Refractory ovarian carcinoma; peritoneal mesothelioma	Ph1, NCT03608618	*Ex vivo* modified macrophages; intraperitoneal infusion	Intraperitoneal CAR-M	Early clinical safety shown; feasibility	Ongoing/early
CAR- monocyte	MT-101 (anti-CD5) / Myeloid Therapeutics	Peripheral T-cell lymphoma	Ph1/2, NCT05138458	*Ex vivo* modified monocytes; reinfused IV	Autologous CAR-monocytes	First-in-human; dosing started 2022	Ongoing
TLR9 agonist (intratumoral)	Tilsotolimod	PD-1-refractory melanoma	Ph3 ILLUMINATE-301, NCT03445533	Intratumoral injections	+ ipilimumab vs ipilimumab	No benefit in ORR/OS	Negative
TLR9 agonist (intratumoral)	Vidutolimod (CMP-001)	High-risk resectable melanoma (neoadj.)	Ph2, NCT04401995	Intratumoral injections + systemic anti-PD-1	+ nivolumab	55% MPR^13^; met primary endpoint	Ongoing / expansion

^1^Gastroesophageal junction. ^2^Quality of Life. ^3^Diffuse tenosynovial giant cell tumor. ^4^Triple negative breast cancer. ^5^Overall Survival. ^6^Metastatic pancreatic ductal adenocarcinoma. ^7^Confirmed Objective Response Rate. ^8^Median Progression-Free Survival. ^9^Median Overall Survival. ^10^Platinum resistant ovarian cancer. ^11^Pharmacokinetics; Receptor Occupancy. ^12^Treatment-related adverse events. ^13^Major pathological response.

## Glossary

AMs: alveolar macrophages
BsAbs: bispecific antibodies
CAR(s): chimeric antigen receptor(s)
CAR-M/T: chimeric antigen receptor macrophages / T-cells
CCL2/3/5/20/26: C-C motif chemokine ligand 2/3/5/20/26
CR1/5/6: C-C motif chemokine receptor type 1/5/6
circRNA: circular RNA
CSF-1(R): colony-stimulating factor 1 (receptor)
CX3CL1: CX3C chemokine ligand 1
CX3CR1: CX3C motif chemokine receptor 1
CXCL9/10/11/12: C-X-C motif chemokine ligand 9/10/11/12
DNMT: DNA methyltransferase
ECM: extracellular matrix
EGF: epidermal growth factor
EMT: epithelial-mesenchymal transition
EVs: extracellular vesicles
EZH2: enhancer of zeste homolog 2
FAO: fatty acid oxidation
FCN1: ficolin-1
GBM: glioblastoma
GM-CSF: granulocyte-macrophage colony-stimulating factor
HDAC: histone deacetylase
HIF-1α/2α: hypoxia inducible factor-1α/2α
ICIs: immune checkpoint inhibitors
IFN(-β/-γ): interferon(-β/-γ)
IL-4/10/34: interleukin-4/10/34
LNPs: lipid nanoparticles
LPMs: large peritoneal macrophages
LYVE-1: lymphatic vessel endothelial receptor 1
MARCO: macrophage receptor with collagenous structure
MDCs: macrophage-drug conjugates
MDMs: monocyte-derived macrophages
MGTRMs: mammary gland tissue-resident macrophages
MHC I/II: major histocompatibility complex class I/II
miRNA: microRNA
mRNA: messenger RNA
mTORC1: mammalian target of rapamycin complex 1
NK: natural killer cell
NO: nitric oxide
NSCLC: non-small cell lung cancer
ORR: overall response rate
PDAC: pancreatic ductal adenocarcinoma
PD-L1: programmed death-ligand 1
ROS: reactive oxygen species
saRNA: small activating RNA
siRNA: small interfering RNA
STAT3: signal transducer and activator of transcription 3
TAMs: tumor-associated macrophages
TGF-β: transforming growth factor β
TLR (4): Toll-like receptor (4)
TME: tumor microenvironment
TNF-α: tumor necrosis factor α
TRAIL: TNF-related apoptosis-inducing ligand
Treg(s): regulatory T cell(s)
TREM2: triggering receptor expressed on myeloid cells 2
TRMs: tissue-resident macrophages
VEGF-A: vascular endothelial growth factor A
α-KG: α-ketoglutarate

## References

[1] Turlej E Domaradzka A Radzka J Drulis-Fajdasz D Kulbacka J Gizak A. Cross-talk between cancer and its cellular environment—A role in cancer progression. Cells. (2025) 14:403. doi: 10.3390/cells14060403, PMID: 40136652 PMC11940884

[2] Morabito M Thibodot P Gigandet A Compagnon P Toso C Berishvili E. Liver extracellular matrix in colorectal liver metastasis. Cancers. (2025) 17:953. doi: 10.3390/cancers17060953, PMID: 40149289 PMC11939972

[3] Soko GF Kosgei BK Meena SS Ng YJ Liang H Zhang B. Extracellular matrix re-normalization to improve cold tumor penetration by oncolytic viruses. Front Immunol. (2025) 15:1535647. doi: 10.3389/fimmu.2024.1535647, PMID: 39845957 PMC11751056

[4] Ricci JE. Tumor-induced metabolic immunosuppression: Mechanisms and therapeutic targets. Cell Rep. (2025) 44:115206. doi: 10.1016/j.celrep.2024.115206, PMID: 39798090

[5] Akinsipe T Mohamedelhassan R Akinpelu A Pondugula SR Mistriotis P Avila LA. Cellular interactions in tumor microenvironment during breast cancer progression: new frontiers and implications for novel therapeutics. Front Immunol. (2024) 15:1302587. doi: 10.3389/fimmu.2024.1302587, PMID: 38533507 PMC10963559

[6] Peppicelli S Calorini L Bianchini F Papucci L Magnelli L Andreucci E. Acidity and hypoxia of tumor microenvironment, a positive interplay in extracellular vesicle release by tumor cells. Cell Oncol Dordr Neth. (2025) 48:27–41. doi: 10.1007/s13402-024-00969-z, PMID: 39023664 PMC11850579

[7] Belényesi SK Patmore S O’Driscoll L. Extracellular vesicles and the tumour microenvironment. Biochim Biophys Acta Rev Cancer. (2025) 1880:189275. doi: 10.1016/j.bbcan.2025.189275, PMID: 39900204

[8] Semeradtova A Liegertova M Herma R Capkova M Brignole C Del Zotto G. Extracellular vesicles in cancer´s communication: messages we can read and how to answer. Mol Cancer. (2025) 24:86. doi: 10.1186/s12943-025-02282-1, PMID: 40108630 PMC11921637

[9] Garg P Ramisetty SK Raghu Subbalakshmi A Krishna BM Pareek S Mohanty A. Gynecological cancer tumor Microenvironment: Unveiling cellular complexity and therapeutic potential. Biochem Pharmacol. (2024) 229:116498. doi: 10.1016/j.bcp.2024.116498, PMID: 39159874

[10] Wang J Chen Q Shan Q Liang T Forde P Zheng L. Clinical development of immuno-oncology therapeutics. Cancer Lett. (2025) 617:217616. doi: 10.1016/j.canlet.2025.217616, PMID: 40054657 PMC11930610

[11] Xu Q Shao D. Leveraging the synergy between anti-angiogenic therapy and immune checkpoint inhibitors to treat digestive system cancers. Front Immunol. (2024) 15:1487610. doi: 10.3389/fimmu.2024.1487610, PMID: 39691707 PMC11649667

[12] Kouidhi S Ben Ayed F Benammar Elgaaied A. Targeting tumor metabolism: A new challenge to improve immunotherapy. Front Immunol. (2018) 9:353. doi: 10.3389/fimmu.2018.00353, PMID: 29527212 PMC5829092

[13] Shah DD Chorawala MR Raghani NR Patel R Fareed M Kashid VA. Tumor microenvironment: recent advances in understanding and its role in modulating cancer therapies. Med Oncol Northwood Lond Engl. (2025) 42:117. doi: 10.1007/s12032-025-02641-4, PMID: 40102282

[14] Verona F Di Bella S Schirano R Manfredi C Angeloro F Bozzari G. Cancer stem cells and tumor-associated macrophages as mates in tumor progression: mechanisms of crosstalk and advanced bioinformatic tools to dissect their phenotypes and interaction. Front Immunol. (2025) 16:1529847. doi: 10.3389/fimmu.2025.1529847, PMID: 39981232 PMC11839637

[15] Wang D Han X Liu HL. The role and research progress of tumor-associated macrophages in cervical cancer. Am J Cancer Res. (2024) 14:5999–6011. doi: 10.62347/FFXL7288, PMID: 39803646 PMC11711540

[16] Larionova I Tuguzbaeva G Ponomaryova A Stakheyeva M Cherdyntseva N Pavlov V. Tumor-associated macrophages in human breast, colorectal, lung, ovarian and prostate cancers. Front Oncol. (2020) 10:566511. doi: 10.3389/fonc.2020.566511, PMID: 33194645 PMC7642726

[17] Zhao X Qu J Sun Y Wang J Liu X Wang F. Prognostic significance of tumor-associated macrophages in breast cancer: a meta-analysis of the literature. Oncotarget. (2017) 8:30576–86. doi: 10.18632/oncotarget.15736, PMID: 28427165 PMC5444766

[18] Zhang B Yao G Zhang Y Gao J Yang B Rao Z. M2-Polarized tumor-associated macrophages are associated with poor prognoses resulting from accelerated lymphangiogenesis in lung adenocarcinoma. Clinics. (2011) 66:1879–86. doi: 10.1590/S1807-59322011001100006, PMID: 22086517 PMC3203959

[19] Yan Y Zhang J Li JH Liu X Wang JZ Qu HY. High tumor-associated macrophages infiltration is associated with poor prognosis and may contribute to the phenomenon of epithelial-mesenchymal transition in gastric cancer. OncoTargets Ther. (2016) 9:3975–83. doi: 10.2147/OTT.S103112, PMID: 27418840 PMC4935103

[20] Kubota K Moriyama M Furukawa S Rafiul HASM Maruse Y Jinno T. CD163+CD204+ tumor-associated macrophages contribute to T cell regulation via interleukin-10 and PD-L1 production in oral squamous cell carcinoma. Sci Rep. (2017) 7:1755. doi: 10.1038/s41598-017-01661-z, PMID: 28496107 PMC5431876

[21] Debacker JM Gondry O Lahoutte T Keyaerts M Huvenne W. The prognostic value of CD206 in solid Malignancies: A systematic review and meta-analysis. Cancers. (2021) 13:3422. doi: 10.3390/cancers13143422, PMID: 34298638 PMC8305473

[22] Nalio Ramos R Missolo-Koussou Y Gerber-Ferder Y Bromley CP Bugatti M Núñez NG. Tissue-resident FOLR2+ macrophages associate with CD8+ T cell infiltration in human breast cancer. Cell. (2022) 185:1189–1207.e25. doi: 10.1016/j.cell.2022.02.021, PMID: 35325594

[23] Li H Miao Y Zhong L Feng S Xu Y Tang L. Identification of TREM2-positive tumor-associated macrophages in esophageal squamous cell carcinoma: implication for poor prognosis and immunotherapy modulation. Front Immunol. (2023) 14:1162032. doi: 10.3389/fimmu.2023.1162032, PMID: 37187751 PMC10175681

[24] Wang Y Chaffee TS LaRue RS Huggins DN Witschen PM Ibrahim AM. Tissue-resident macrophages promote extracellular matrix homeostasis in the mammary gland stroma of nulliparous mice. eLife. (2020) 9:e57438. doi: 10.7554/eLife.57438.sa2, PMID: 32479261 PMC7297528

[25] Chi J Gao Q Liu D. Tissue-resident macrophages in cancer: friend or foe? Cancer Med. (2024) 13:e70387. doi: 10.1002/cam4.70387, PMID: 39494816 PMC11533131

[26] Wang C Chen L Fu D Liu W Puri A Kellis M. Antigen presenting cells in cancer immunity and mediation of immune checkpoint blockade. Clin Exp Metastasis. (2024) 41:333–49. doi: 10.1007/s10585-023-10257-z, PMID: 38261139 PMC11374820

[27] Verneau J Sautés-Fridman C Sun CM. Dendritic cells in the tumor microenvironment: prognostic and theranostic impact. Semin Immunol. (2020) 48:101410. doi: 10.1016/j.smim.2020.101410, PMID: 33011065

[28] Liu J Geng X Hou J Wu G. New insights into M1/M2 macrophages: key modulators in cancer progression. Cancer Cell Int. (2021) 21:389. doi: 10.1186/s12935-021-02089-2, PMID: 34289846 PMC8296555

[29] Pan Y Yu Y Wang X Zhang T. Tumor-associated macrophages in tumor immunity. Front Immunol. (2020) 11:583084. doi: 10.3389/fimmu.2020.583084, PMID: 33365025 PMC7751482

[30] Qi J Sun H Zhang Y Wang Z Xun Z Li Z. Single-cell and spatial analysis reveal interaction of FAP+ fibroblasts and SPP1+ macrophages in colorectal cancer. Nat Commun. (2022) 13:1742. doi: 10.1038/s41467-022-29366-6, PMID: 35365629 PMC8976074

[31] Liu C Wu K Li C Zhang Z Zhai P Guo H. SPP1+ macrophages promote head and neck squamous cell carcinoma progression by secreting TNF-α and IL-1β. J Exp Clin Cancer Res. (2024) 43:332. doi: 10.1186/s13046-024-03255-w, PMID: 39726047 PMC11670405

[32] Korbecki J Olbromski M Dzięgiel P. CCL18 in the progression of cancer. Int J Mol Sci. (2020) 21:7955. doi: 10.3390/ijms21217955, PMID: 33114763 PMC7663205

[33] Yang Q Zhang H Wei T Lin A Sun Y Luo P. Single-cell RNA sequencing reveals the heterogeneity of tumor-associated macrophage in non-small cell lung cancer and differences between sexes. Front Immunol. (2021) 12:756722. doi: 10.3389/fimmu.2021.756722, PMID: 34804043 PMC8602907

[34] Huang X Lai S Qu F Li Z Fu X Li Q. CCL18 promotes breast cancer progression by exosomal miR-760 activation of ARF6/Src/PI3K/Akt pathway. Mol Ther Oncolytics. (2022) 25:1–15. doi: 10.1016/j.omto.2022.03.004, PMID: 35399607 PMC8971730

[35] Yue T Liu X Zuo S Zhu J Li J Liu Y. BCL2A1 and CCL18 are predictive biomarkers of cisplatin chemotherapy and immunotherapy in colon cancer patients. Front Cell Dev Biol. (2021) 9:799278. doi: 10.3389/fcell.2021.799278, PMID: 35265629 PMC8898943

[36] Chu T Zhu G Tang Z Qu W Yang R Pan H. Metabolism archetype cancer cells induce protumor TREM2+ macrophages via oxLDL-mediated metabolic interplay in hepatocellular carcinoma. Nat Commun. (2025) 16:6770. doi: 10.1038/s41467-025-62132-y, PMID: 40695827 PMC12284189

[37] Tan J Fan W Liu T Zhu B Liu Y Wang S. TREM2+ macrophages suppress CD8+ T-cell infiltration after transarterial chemoembolisation in hepatocellular carcinoma. J Hepatol. (2023) 79:126–40. doi: 10.1016/j.jhep.2023.02.032, PMID: 36889359

[38] Zhang Q He Y Luo N Patel SJ Han Y Gao R. Landscape and dynamics of single immune cells in hepatocellular carcinoma. Cell. (2019) 179:829–845.e20. doi: 10.1016/j.cell.2019.10.003, PMID: 31675496

[39] Zhang L Li Z Skrzypczynska KM Fang Q Zhang W O’Brien SA. Single-cell analyses inform mechanisms of myeloid-targeted therapies in colon cancer. Cell. (2020) 181:442–459.e29. doi: 10.1016/j.cell.2020.03.048, PMID: 32302573

[40] Ammarah U Pereira-Nunes A Delfini M Mazzone M. From monocyte-derived macrophages to resident macrophages—how metabolism leads their way in cancer. Mol Oncol. (2024) 18:1739–58. doi: 10.1002/1878-0261.13618, PMID: 38411356 PMC11223613

[41] Cortez-Retamozo V Etzrodt M Newton A Rauch PJ Chudnovskiy A Berger C. Origins of tumor-associated macrophages and neutrophils. Proc Natl Acad Sci U S A. (2012) 109:2491–6. doi: 10.1073/pnas.1113744109, PMID: 22308361 PMC3289379

[42] Casanova-Acebes M Dalla E Leader AM LeBerichel J Nikolic J Morales BM. Tissue-resident macrophages provide a pro-tumorigenic niche to early NSCLC cells. Nature. (2021) 595:578–84. doi: 10.1038/s41586-021-03651-8, PMID: 34135508 PMC8923521

[43] Andersen RS Anand A Harwood DSL Kristensen BW. Tumor-associated microglia and macrophages in the glioblastoma microenvironment and their implications for therapy. Cancers. (2021) 13:4255. doi: 10.3390/cancers13174255, PMID: 34503065 PMC8428223

[44] Wu K Kryczek I Chen L Zou W Welling TH. Kupffer cell suppression of CD8+ T cells in human hepatocellular carcinoma is mediated by B7-H1/PD-1 interactions. Cancer Res. (2009) 69:8067–75. doi: 10.1158/0008-5472.CAN-09-0901, PMID: 19826049 PMC4397483

[45] Li T Song X Chen J Li Y Lin J Li P. Kupffer cell-derived IL6 promotes hepatocellular carcinoma metastasis via the JAK1-ACAP4 pathway. Int J Biol Sci. (2025) 21:285–305. doi: 10.7150/ijbs.97109, PMID: 39744421 PMC11667824

[46] Li X Li R Miao X Zhou X Wu B Cao J. Integrated single cell analysis reveals an atlas of tumor associated macrophages in hepatocellular carcinoma. Inflammation. (2024) 47:2077–93. doi: 10.1007/s10753-024-02026-1, PMID: 38668836

[47] Huang S He L Zhao Y Wei Y Wang Q Gao Y. TREM1+ tumor-associated macrophages secrete CCL7 to promote hepatocellular carcinoma metastasis. J Cancer Res Clin Oncol. (2024) 150:320. doi: 10.1007/s00432-024-05831-1, PMID: 38914803 PMC11196310

[48] Liu S Zhang S Dong H Jin X Sun J Zhou H. CD63 + tumor-associated macrophages drive the progression of hepatocellular carcinoma through the induction of epithelial-mesenchymal transition and lipid reprogramming. BMC Cancer. (2024) 24:698. doi: 10.1186/s12885-024-12472-7, PMID: 38849760 PMC11157766

[49] Zhu Y Herndon JM Sojka DK Kim KW Knolhoff BL Zuo C. Tissue-resident macrophages in pancreatic ductal adenocarcinoma originate from embryonic hematopoiesis and promote tumor progression. Immunity. (2017) 47:597. doi: 10.1016/j.immuni.2017.08.018, PMID: 28930665 PMC5664180

[50] Yang S Liu Q Liao Q. Tumor-associated macrophages in pancreatic ductal adenocarcinoma: origin, polarization, function, and reprogramming. Front Cell Dev Biol. (2021) 8:607209. doi: 10.3389/fcell.2020.607209, PMID: 33505964 PMC7829544

[51] Hirano R Okamoto K Shinke M Sato M Watanabe S Watanabe H. Tissue-resident macrophages are major tumor-associated macrophage resources, contributing to early TNBC development, recurrence, and metastases. Commun Biol. (2023) 6:144. doi: 10.1038/s42003-023-04525-7, PMID: 36737474 PMC9898263

[52] Etzerodt A Moulin M Doktor TK Delfini M Mossadegh-Keller N Bajenoff M. Tissue-resident macrophages in omentum promote metastatic spread of ovarian cancer. J Exp Med. (2020) 217:e20191869. doi: 10.1084/jem.20191869, PMID: 31951251 PMC7144521

[53] Matusiak M Hickey JW van IJzendoorn DGP Lu G Kidziński L Zhu S. Spatially segregated macrophage populations predict distinct outcomes in colon cancer. Cancer Discov. (2024) 14:1418–39. doi: 10.1158/2159-8290.CD-23-1300, PMID: 38552005 PMC11294822

[54] Kzhyshkowska J Shen J Larionova I. Targeting of TAMs: can we be more clever than cancer cells? Cell Mol Immunol. (2024) 21:1376–409. doi: 10.1038/s41423-024-01232-z, PMID: 39516356 PMC11607358

[55] Lv T Fan R Wu J Gong H Gao X Liu X. Tumor-associated macrophages: key players in the non-small cell lung cancer tumor microenvironment. Cancer Med. (2025) 14:e70670. doi: 10.1002/cam4.70670, PMID: 39927632 PMC11808749

[56] Murdoch C Giannoudis A Lewis CE. Mechanisms regulating the recruitment of macrophages into hypoxic areas of tumors and other ischemic tissues. Blood. (2004) 104:2224–34. doi: 10.1182/blood-2004-03-1109, PMID: 15231578

[57] Stafford JH Hirai T Deng L Chernikova SB Urata K West BL. Colony stimulating factor 1 receptor inhibition delays recurrence of glioblastoma after radiation by altering myeloid cell recruitment and polarization. Neuro-Oncol. (2016) 18:797–806. doi: 10.1093/neuonc/nov272, PMID: 26538619 PMC4864255

[58] Qian BZ Li J Zhang H Kitamura T Zhang J Campion LR. CCL2 recruits inflammatory monocytes to facilitate breast-tumour metastasis. Nature. (2011) 475:222–5. doi: 10.1038/nature10138, PMID: 21654748 PMC3208506

[59] Tomassetti C Insinga G Gimigliano F Morrione A Giordano A Giurisato E. Insights into CSF-1R expression in the tumor microenvironment. Biomedicines. (2024) 12:2381. doi: 10.3390/biomedicines12102381, PMID: 39457693 PMC11504891

[60] Blondy T d’Almeida SM Briolay T Tabiasco J Meiller C Chéné AL. Involvement of the M-CSF/IL-34/CSF-1R pathway in Malignant pleural mesothelioma. J Immunother Cancer. (2020) 8:e000182. doi: 10.1136/jitc-2019-000182, PMID: 32581053 PMC7319783

[61] Nian Z Dou Y Shen Y Liu J Du X Jiang Y. Interleukin-34-orchestrated tumor-associated macrophage reprogramming is required for tumor immune escape driven by p53 inactivation. Immunity. (2024) 57:2344–2361.e7. doi: 10.1016/j.immuni.2024.08.015, PMID: 39321806

[62] Korbecki J Simińska D Kojder K Grochans S Gutowska I Chlubek D. Fractalkine/CX3CL1 in neoplastic processes. Int J Mol Sci. (2020) 21:3723. doi: 10.3390/ijms21103723, PMID: 32466280 PMC7279446

[63] Ishida Y Kuninaka Y Yamamoto Y Nosaka M Kimura A Furukawa F. Pivotal involvement of the CX3CL1-CX3CR1 axis for the recruitment of M2 tumor-associated macrophages in skin carcinogenesis. J Invest Dermatol. (2020) 140:1951–1961.e6. doi: 10.1016/j.jid.2020.02.023, PMID: 32179066

[64] Le K Sun J Ghaemmaghami J Smith MR Ip WKE Phillips T. Blockade of CCR1 induces a phenotypic shift in macrophages and triggers a favorable antilymphoma activity. Blood Adv. (2023) 7:3952–67. doi: 10.1182/bloodadvances.2022008722, PMID: 36630565 PMC10410136

[65] Qin R Ren W Ya G Wang B He J Ren S. Role of chemokines in the crosstalk between tumor and tumor-associated macrophages. Clin Exp Med. (2023) 23:1359–73. doi: 10.1007/s10238-022-00888-z, PMID: 36173487 PMC10460746

[66] Aldinucci D Borghese C Casagrande N. The CCL5/CCR5 axis in cancer progression. Cancers. (2020) 12:1765. doi: 10.3390/cancers12071765, PMID: 32630699 PMC7407580

[67] Nandi B Pai C Huang Q Prabhala RH Munshi NC Gold JS. CCR6, the sole receptor for the chemokine CCL20, promotes spontaneous intestinal tumorigenesis. PloS One. (2014) 9:e97566. doi: 10.1371/journal.pone.0097566, PMID: 24866282 PMC4035256

[68] Li C Liu B Dai Z Tao Y. Knockdown of VEGF receptor-1 (VEGFR-1) impairs macrophage infiltration, angiogenesis and growth of clear cell renal cell carcinoma (CRCC). Cancer Biol Ther. (2011) 12:872–80. doi: 10.4161/cbt.12.10.17672, PMID: 21989163 PMC3280902

[69] Muñoz-Garcia J Cochonneau D Télétchéa S Moranton E Lanoe D Brion R. The twin cytokines interleukin-34 and CSF-1: masterful conductors of macrophage homeostasis. Theranostics. (2021) 11:1568–93. doi: 10.7150/thno.50683, PMID: 33408768 PMC7778581

[70] Sun X Wang X Yan C Zheng S Gao R Huang F. Tumor cell-released LC3-positive EVs promote lung metastasis of breast cancer through enhancing premetastatic niche formation. Cancer Sci. (2022) 113:3405–16. doi: 10.1111/cas.15507, PMID: 35879596 PMC9530874

[71] Zhang Y Wang X Gu Y Liu T Zhao X Cheng S. Complement C3 of tumor-derived extracellular vesicles promotes metastasis of RCC via recruitment of immunosuppressive myeloid cells. Proc Natl Acad Sci U S A. (2025) 122:e2420005122. doi: 10.1073/pnas.2420005122, PMID: 39847320 PMC11789090

[72] Lima LG Ham S Shin H Chai EPZ Lek ESH Lobb RJ. Tumor microenvironmental cytokines bound to cancer exosomes determine uptake by cytokine receptor-expressing cells and biodistribution. Nat Commun. (2021) 12:3543. doi: 10.1038/s41467-021-23946-8, PMID: 34112803 PMC8192925

[73] Baj-Krzyworzeka M Szatanek R Weglarczyk K Baran J Urbanowicz B Brański P. Tumour-derived microvesicles carry several surface determinants and mRNA of tumour cells and transfer some of these determinants to monocytes. Cancer Immunol Immunother CII. (2006) 55:808–18. doi: 10.1007/s00262-005-0075-9, PMID: 16283305 PMC11030663

[74] Luong N Lenz JA Modiano JF Olson JK. Extracellular vesicles secreted by tumor cells promote the generation of suppressive monocytes. ImmunoHorizons. (2021) 5:647–58. doi: 10.4049/immunohorizons.2000017, PMID: 34404719

[75] Lenart M Rutkowska-Zapala M Baj-Krzyworzeka M Szatanek R Węglarczyk K Smallie T. Hyaluronan carried by tumor-derived microvesicles induces IL-10 production in classical (CD14++CD16-) monocytes via PI3K/Akt/mTOR-dependent signalling pathway. Immunobiology. (2017) 222:1–10. doi: 10.1016/j.imbio.2015.06.019, PMID: 26210045

[76] Hummitzsch L Berndt R Kott M Rusch R Faendrich F Gruenewald M. Hypoxia directed migration of human naïve monocytes is associated with an attenuation of cytokine release: indications for a key role of CCL26. J Transl Med. (2020) 18:404. doi: 10.1186/s12967-020-02567-7, PMID: 33087148 PMC7579884

[77] Wang SC Hong JH Hsueh C Chiang CS. Tumor-secreted SDF-1 promotes glioma invasiveness and TAM tropism toward hypoxia in a murine astrocytoma model. Lab Investig J Tech Methods Pathol. (2012) 92:151–62. doi: 10.1038/labinvest.2011.128, PMID: 21894147

[78] Henze AT Mazzone M. The impact of hypoxia on tumor-associated macrophages. J Clin Invest. (2016) 126:3672–9. doi: 10.1172/JCI84427, PMID: 27482883 PMC5096805

[79] Sun K Zhang X Shi J Huang J Wang S Li X. Elevated protein lactylation promotes immunosuppressive microenvironment and therapeutic resistance in pancreatic ductal adenocarcinoma. J Clin Invest. (2025) 135:e187024. doi: 10.1172/JCI187024, PMID: 39883522 PMC11957693

[80] Wang SC Yu CF Hong JH Tsai CS Chiang CS. Radiation therapy-induced tumor invasiveness is associated with SDF-1-regulated macrophage mobilization and vasculogenesis. PloS One. (2013) 8:e69182. doi: 10.1371/journal.pone.0069182, PMID: 23940516 PMC3734136

[81] Baghdadi M Wada H Nakanishi S Abe H Han N Putra WE. Chemotherapy-induced IL34 enhances immunosuppression by tumor-associated macrophages and mediates survival of chemoresistant lung cancer cells. Cancer Res. (2016) 76:6030–42. doi: 10.1158/0008-5472.CAN-16-1170, PMID: 27550451

[82] Han N Baghdadi M Ishikawa K Endo H Kobayashi T Wada H. Enhanced IL-34 expression in Nivolumab-resistant metastatic melanoma. Inflammation Regen. (2018) 38:3. doi: 10.1186/s41232-018-0060-2, PMID: 29515691 PMC5836392

[83] Boulakirba S Pfeifer A Mhaidly R Obba S Goulard M Schmitt T. IL-34 and CSF-1 display an equivalent macrophage differentiation ability but a different polarization potential. Sci Rep. (2018) 8:256. doi: 10.1038/s41598-017-18433-4, PMID: 29321503 PMC5762882

[84] Makita N Hizukuri Y Yamashiro K Murakawa M Hayashi Y. IL-10 enhances the phenotype of M2 macrophages induced by IL-4 and confers the ability to increase eosinophil migration. Int Immunol. (2015) 27:131–41. doi: 10.1093/intimm/dxu090, PMID: 25267883

[85] Kim HJ Park JH Kim HC Kim CW Kang I Lee HK. Blood monocyte-derived CD169+ macrophages contribute to antitumor immunity against glioblastoma. Nat Commun. (2022) 13:6211. doi: 10.1038/s41467-022-34001-5, PMID: 36266311 PMC9585054

[86] Ji ZZ Chan MKK Chan ASW Leung KT Jiang X To KF. Tumour-associated macrophages: versatile players in the tumour microenvironment. Front Cell Dev Biol. (2023) 11:1261749. doi: 10.3389/fcell.2023.1261749, PMID: 37965573 PMC10641386

[87] Bied M Ho WW Ginhoux F Blériot C. Roles of macrophages in tumor development: a spatiotemporal perspective. Cell Mol Immunol. (2023) 20:983–92. doi: 10.1038/s41423-023-01061-6, PMID: 37429944 PMC10468537

[88] Hwang I Kim JW Ylaya K Chung EJ Kitano H Perry C. Tumor-associated macrophage, angiogenesis and lymphangiogenesis markers predict prognosis of non-small cell lung cancer patients. J Transl Med. (2020) 18:443. doi: 10.1186/s12967-020-02618-z, PMID: 33228719 PMC7686699

[89] Yang Y Li S To KKW Zhu S Wang F Fu L. Tumor-associated macrophages remodel the suppressive tumor immune microenvironment and targeted therapy for immunotherapy. J Exp Clin Cancer Res. (2025) 44:145. doi: 10.1186/s13046-025-03377-9, PMID: 40380196 PMC12083052

[90] Huang L Yang J Zhu J Wang H Dong L Guo Y. Programmed death ligand-1 in melanoma and extracellular vesicles promotes local and regional immune suppression through M2-like macrophage polarization. Am J Pathol. (2025) 195:306–20. doi: 10.1016/j.ajpath.2024.09.011, PMID: 39481645 PMC11773617

[91] Friedman-DeLuca M Karagiannis GS Condeelis JS Oktay MH Entenberg D. Macrophages in tumor cell migration and metastasis. Front Immunol. (2024) 15:1494462. doi: 10.3389/fimmu.2024.1494462, PMID: 39555068 PMC11563815

[92] Guo S Chen X Guo C Wang W. Tumour-associated macrophages heterogeneity drives resistance to clinical therapy. Expert Rev Mol Med. (2022) 24:e17. doi: 10.1017/erm.2022.8, PMID: 35400355 PMC9884773

[93] Jang HJ Lee HS Yu W Ramineni M Truong CY Ramos D. Therapeutic targeting of macrophage plasticity remodels the tumor-immune microenvironment. Cancer Res. (2022) 82:2593–609. doi: 10.1158/0008-5472.CAN-21-3506, PMID: 35709756 PMC9296613

[94] Nobs SP Kopf M. Tissue-resident macrophages: guardians of organ homeostasis. Trends Immunol. (2021) 42:495–507. doi: 10.1016/j.it.2021.04.007, PMID: 33972166

[95] Lazarov T Juarez-Carreño S Cox N Geissmann F. Physiology and diseases of tissue-resident macrophages. Nature. (2023) 618:698–707. doi: 10.1038/s41586-023-06002-x, PMID: 37344646 PMC10649266

[96] Ham S Lima LG Lek E Möller A. The impact of the cancer microenvironment on macrophage phenotypes. Front Immunol. (2020) 11:1308. doi: 10.3389/fimmu.2020.01308, PMID: 32655574 PMC7324670

[97] Samaniego R Domínguez-Soto Á Ratnam M Matsuyama T Sánchez-Mateos P Corbí ÁL. Folate receptor β (FRβ) expression in tissue-resident and tumor-associated macrophages associates with and depends on the expression of PU.1. Cells. (2020) 9:1445. doi: 10.3390/cells9061445, PMID: 32532019 PMC7349916

[98] Rojo R Raper A Ozdemir DD Lefevre L Grabert K Wollscheid-Lengeling E. Deletion of a Csf1r enhancer selectively impacts CSF1R expression and development of tissue macrophage populations. Nat Commun. (2019) 10:3215. doi: 10.1038/s41467-019-11053-8, PMID: 31324781 PMC6642117

[99] Biswas M. Understanding tissue-resident macrophages unlocks the potential for novel combinatorial strategies in breast cancer. Front Immunol. (2024) 15:1375528. doi: 10.3389/fimmu.2024.1375528, PMID: 39104525 PMC11298421

[100] Loyher PL Hamon P Laviron M Meghraoui-Kheddar A Goncalves E Deng Z. Macrophages of distinct origins contribute to tumor development in the lung. J Exp Med. (2018) 215:2536–53. doi: 10.1084/jem.20180534, PMID: 30201786 PMC6170177

[101] Prieto LI Sturmlechner I Graves SI Zhang C Goplen NP Yi ES. Senescent alveolar macrophages promote early-stage lung tumorigenesis. Cancer Cell. (2023) 41:1261–1275.e6. doi: 10.1016/j.ccell.2023.05.006, PMID: 37267954 PMC10524974

[102] Pouniotis DS Plebanski M Apostolopoulos V McDonald CF. Alveolar macrophage function is altered in patients with lung cancer. Clin Exp Immunol. (2006) 143:363–72. doi: 10.1111/j.1365-2249.2006.02998.x, PMID: 16412062 PMC1809587

[103] Taniguchi S Matsui T Kimura K Funaki S Miyamoto Y Uchida Y. *In vivo* induction of activin A-producing alveolar macrophages supports the progression of lung cell carcinoma. Nat Commun. (2023) 14:143. doi: 10.1038/s41467-022-35701-8, PMID: 36650150 PMC9845242

[104] Huang H Yang Y Zhang Q Yang Y Xiong Z Mao S. S100a4+ alveolar macrophages accelerate the progression of precancerous atypical adenomatous hyperplasia by promoting the angiogenic function regulated by fatty acid metabolism. eLife. (2025) 13:RP101731. doi: 10.7554/eLife.101731, PMID: 40658605 PMC12259021

[105] Alikhanyan K Chen Y Kraut S Sotillo R. Targeting alveolar macrophages shows better treatment response than deletion of interstitial macrophages in EGFR mutant lung adenocarcinoma. Immun Inflammation Dis. (2020) 8:181–7. doi: 10.1002/iid3.293, PMID: 32125091 PMC7212197

[106] Jiang X Wang J Lin L Du L Ding Y Zheng F. Macrophages promote pre-metastatic niche formation of breast cancer through aryl hydrocarbon receptor activity. Signal Transduct Target Ther. (2024) 9:352. doi: 10.1038/s41392-024-02042-5, PMID: 39690159 PMC11652640

[107] Ji H Houghton AM Mariani TJ Perera S Kim CB Padera R. K-ras activation generates an inflammatory response in lung tumors. Oncogene. (2006) 25:2105–12. doi: 10.1038/sj.onc.1209237, PMID: 16288213

[108] Kramer ED Tzetzo SL Colligan SH Hensen ML Brackett CM Clausen BE. β-Catenin signaling in alveolar macrophages enhances lung metastasis through a TNF-dependent mechanism. JCI Insight. (2023) 8:e160978. doi: 10.1172/jci.insight.160978, PMID: 37092550 PMC10243816

[109] Nosaka T Baba T Tanabe Y Sasaki S Nishimura T Imamura Y. Alveolar macrophages drive hepatocellular carcinoma lung metastasis by generating leukotriene B4. J Immunol Baltim Md. (1950) 200:1839–52. doi: 10.4049/jimmunol.1700544, PMID: 29378914

[110] Sharma SK Chintala NK Vadrevu SK Patel J Karbowniczek M Markiewski MM. Pulmonary alveolar macrophages contribute to the premetastatic niche by suppressing antitumor T cell responses in the lungs. J Immunol Baltim Md. (1950) 194:5529–38. doi: 10.4049/jimmunol.1403215, PMID: 25911761

[111] Almatroodi SA McDonald CF Pouniotis DS. Alveolar macrophage polarisation in lung cancer. Lung Cancer Int. (2014) 2014:721087. doi: 10.1155/2014/721087, PMID: 26316944 PMC4437403

[112] Hussain SF Yang D Suki D Aldape K Grimm E Heimberger AB. The role of human glioma-infiltrating microglia/macrophages in mediating antitumor immune responses. Neuro-Oncol. (2006) 8:261–79. doi: 10.1215/15228517-2006-008, PMID: 16775224 PMC1871955

[113] Maas SLN Abels ER Van De Haar LL Zhang X Morsett L Sil S. Glioblastoma hijacks microglial gene expression to support tumor growth. J Neuroinflammation. (2020) 17:120. doi: 10.1186/s12974-020-01797-2, PMID: 32299465 PMC7164149

[114] Hambardzumyan D Gutmann DH Kettenmann H. The role of microglia and macrophages in glioma maintenance and progression. Nat Neurosci. (2016) 19:20–7. doi: 10.1038/nn.4185, PMID: 26713745 PMC4876023

[115] Chen Z Feng X Herting CJ Garcia VA Nie K Pong WW. Cellular and molecular identity of tumor-associated macrophages in glioblastoma. Cancer Res. (2017) 77:2266–78. doi: 10.1158/0008-5472.CAN-16-2310, PMID: 28235764 PMC5741820

[116] Coniglio SJ Eugenin E Dobrenis K Stanley ER West BL Symons MH. Microglial stimulation of glioblastoma invasion involves epidermal growth factor receptor (EGFR) and colony stimulating factor 1 receptor (CSF-1R) signaling. Mol Med Camb Mass. (2012) 18:519–27. doi: 10.2119/molmed.2011.00217, PMID: 22294205 PMC3356419

[117] Ku MC Wolf SA Respondek D Matyash V Pohlmann A Waiczies S. GDNF mediates glioblastoma-induced microglia attraction but not astrogliosis. Acta Neuropathol (Berl). (2013) 125:609–20. doi: 10.1007/s00401-013-1079-8, PMID: 23344256

[118] Zhang J Sarkar S Cua R Zhou Y Hader W Yong VW. A dialog between glioma and microglia that promotes tumor invasiveness through the CCL2/CCR2/interleukin-6 axis. Carcinogenesis. (2012) 33:312–9. doi: 10.1093/carcin/bgr289, PMID: 22159219

[119] Wu A Wei J Kong LY Wang Y Priebe W Qiao W. Glioma cancer stem cells induce immunosuppressive macrophages/microglia. Neuro-Oncol. (2010) 12:1113–25. doi: 10.1093/neuonc/noq082, PMID: 20667896 PMC3098021

[120] Yang Y Cui H Li D Chen L Liu Y Zhou C. S100A8 promotes tumor progression by inducing phenotypic polarization of microglia through the TLR4/IL-10 signaling pathway in glioma. J Natl Cancer Cent. (2024) 4:369–81. doi: 10.1016/j.jncc.2024.07.001, PMID: 39735438 PMC11674433

[121] Qian J Luo F Yang J Liu J Liu R Wang L. TLR2 promotes glioma immune evasion by downregulating MHC class II molecules in microglia. Cancer Immunol Res. (2018) 6:1220–33. doi: 10.1158/2326-6066.CIR-18-0020, PMID: 30131377

[122] Hu F Dzaye ODA Hahn A Yu Y Scavetta RJ Dittmar G. Glioma-derived versican promotes tumor expansion via glioma-associated microglial/macrophages Toll-like receptor 2 signaling. Neuro-Oncol. (2015) 17:200–10. doi: 10.1093/neuonc/nou324, PMID: 25452390 PMC4288527

[123] Matias D Dubois LG Pontes B Rosário L Ferrer VP Balça-Silva J. GBM-derived wnt3a induces M2-like phenotype in microglial cells through wnt/β-catenin signaling. Mol Neurobiol. (2019) 56:1517–30. doi: 10.1007/s12035-018-1150-5, PMID: 29948952

[124] Zheng H Jia L Liu CC Rong Z Zhong L Yang L. TREM2 promotes microglial survival by activating wnt/β-catenin pathway. J Neurosci. (2017) 37:1772–84. doi: 10.1523/JNEUROSCI.2459-16.2017, PMID: 28077724 PMC5320608

[125] Komohara Y Ohnishi K Kuratsu J Takeya M. Possible involvement of the M2 anti-inflammatory macrophage phenotype in growth of human gliomas. J Pathol. (2008) 216:15–24. doi: 10.1002/path.2370, PMID: 18553315

[126] Nijaguna MB Patil V Urbach S Shwetha SD Sravani K Hegde AS. Glioblastoma-derived macrophage colony-stimulating factor (MCSF) induces microglial release of insulin-like growth factor-binding protein 1 (IGFBP1) to promote angiogenesis. J Biol Chem. (2015) 290:23401–15. doi: 10.1074/jbc.M115.664037, PMID: 26245897 PMC4645610

[127] De I Steffen MD Clark PA Patros CJ Sokn E Bishop SM. CSF1 overexpression promotes high-grade glioma formation without impacting the polarization status of glioma-associated microglia and macrophages. Cancer Res. (2016) 76:2552–60. doi: 10.1158/0008-5472.CAN-15-2386, PMID: 27013192 PMC4873447

[128] Platten M Kretz A Naumann U Aulwurm S Egashira K Isenmann S. Monocyte chemoattractant protein-1 increases microglial infiltration and aggressiveness of gliomas. Ann Neurol. (2003) 54:388–92. doi: 10.1002/ana.10679, PMID: 12953273

[129] Guo X Xue H Shao Q Wang J Guo X Chen X. Hypoxia promotes glioma-associated macrophage infiltration via periostin and subsequent M2 polarization by upregulating TGF-beta and M-CSFR. Oncotarget. (2016) 7:80521–42. doi: 10.18632/oncotarget.11825, PMID: 27602954 PMC5348338

[130] Liao Y Luo Z Lin Y Chen H Chen T Xu L. PRMT3 drives glioblastoma progression by enhancing HIF1A and glycolytic metabolism. Cell Death Dis. (2022) 13:943. doi: 10.1038/s41419-022-05389-1, PMID: 36351894 PMC9646854

[131] van der Vos KE Abels ER Zhang X Lai C Carrizosa E Oakley D. Directly visualized glioblastoma-derived extracellular vesicles transfer RNA to microglia/macrophages in the brain. Neuro-Oncol. (2016) 18:58–69. doi: 10.1093/neuonc/nov244, PMID: 26433199 PMC4677420

[132] Abels ER Maas SLN Nieland L Wei Z Cheah PS Tai E. Glioblastoma-associated microglia reprogramming is mediated by functional transfer of extracellular miR-21. Cell Rep. (2019) 28:3105–3119.e7. doi: 10.1016/j.celrep.2019.08.036, PMID: 31533034 PMC6817978

[133] Chen T Liu J Wang C Wang Z Zhou J Lin J. ALOX5 contributes to glioma progression by promoting 5-HETE-mediated immunosuppressive M2 polarization and PD-L1 expression of glioma-associated microglia/macrophages. J Immunother Cancer. (2024) 12:e009492. doi: 10.1136/jitc-2024-009492, PMID: 39142719 PMC11332009

[134] Wang XP Guo W Chen YF Hong C Ji J Zhang XY. PD-1/PD-L1 axis is involved in the interaction between microglial polarization and glioma. Int Immunopharmacol. (2024) 133:112074. doi: 10.1016/j.intimp.2024.112074, PMID: 38615383

[135] Yang K Yang T Yu J Li F Zhao X. Integrated transcriptional analysis reveals macrophage heterogeneity and macrophage-tumor cell interactions in the progression of pancreatic ductal adenocarcinoma. BMC Cancer. (2023) 23:199. doi: 10.1186/s12885-023-10675-y, PMID: 36864399 PMC9983236

[136] Baer JM Zuo C Kang LI de la Lastra AA Borcherding NC Knolhoff BL. Fibrosis induced by resident macrophages has divergent roles in pancreas inflammatory injury and PDAC. Nat Immunol. (2023) 24:1443–57. doi: 10.1038/s41590-023-01579-x, PMID: 37563309 PMC10757749

[137] Tandon M Coudriet GM Criscimanna A Socorro M Eliliwi M Singhi AD. Prolactin promotes fibrosis and pancreatic cancer progression. Cancer Res. (2019) 79:5316–27. doi: 10.1158/0008-5472.CAN-18-3064, PMID: 31395607 PMC6801092

[138] Zhang J Song J Tang S Zhao Y Wang L Luo Y. Multi-omics analysis reveals the chemoresistance mechanism of proliferating tissue-resident macrophages in PDAC via metabolic adaptation. Cell Rep. (2023) 42:112620. doi: 10.1016/j.celrep.2023.112620, PMID: 37285267

[139] Zuo C Baer JM Knolhoff BL Belle JI Liu X Hogg GD. Macrophage proliferation machinery leads to PDAC progression, but susceptibility to innate immunotherapy. bioRxiv. (2021). doi: 10.1101/2021.11.08.467770v1 PMC10072222 36951731

[140] Gupta V Yull F Khabele D. Bipolar tumor-associated macrophages in ovarian cancer as targets for therapy. Cancers. (2018) 10:366. doi: 10.3390/cancers10100366, PMID: 30274280 PMC6210537

[141] Miyamoto T Murphy B Zhang N. Intraperitoneal metastasis of ovarian cancer: new insights on resident macrophages in the peritoneal cavity. Front Immunol. (2023) 14:1104694. doi: 10.3389/fimmu.2023.1104694, PMID: 37180125 PMC10167029

[142] Krishnan V Tallapragada S Schaar B Kamat K Chanana AM Zhang Y. Omental macrophages secrete chemokine ligands that promote ovarian cancer colonization of the omentum via CCR1. Commun Biol. (2020) 3:524. doi: 10.1038/s42003-020-01246-z, PMID: 32963283 PMC7508838

[143] Li H Zeng C Shu C Cao Y Shao W Zhang M. Laminins in tumor-derived exosomes upregulated by ETS1 reprogram omental macrophages to promote omental metastasis of ovarian cancer. Cell Death Dis. (2022) 13:1028. doi: 10.1038/s41419-022-05472-7, PMID: 36477408 PMC9729302

[144] Goossens P Rodriguez-Vita J Etzerodt A Masse M Rastoin O Gouirand V. Membrane cholesterol efflux drives tumor-associated macrophage reprogramming and tumor progression. Cell Metab. (2019) 29:1376–1389.e4. doi: 10.1016/j.cmet.2019.02.016, PMID: 30930171

[145] Casanova-Acebes M Menéndez-Gutiérrez MP Porcuna J Álvarez-Errico D Lavin Y García A. RXRs control serous macrophage neonatal expansion and identity and contribute to ovarian cancer progression. Nat Commun. (2020) 11:1655. doi: 10.1038/s41467-020-15371-0, PMID: 32246014 PMC7125161

[146] Xia H Li S Li X Wang W Bian Y Wei S. Autophagic adaptation to oxidative stress alters peritoneal residential macrophage survival and ovarian cancer metastasis. JCI Insight. (2020) 5:e141115. doi: 10.1172/jci.insight.141115, PMID: 32780724 PMC7526547

[147] Joshi S López L Morosi LG Amadio R Pachauri M Bestagno M. Tim4 enables large peritoneal macrophages to cross-present tumor antigens at early stages of tumorigenesis. Cell Rep. (2024) 43:114096. doi: 10.1016/j.celrep.2024.114096, PMID: 38607919

[148] Wu Y Teh YC Chong SZ. Going full teRM: the seminal role of tissue-resident macrophages in organ remodeling during pregnancy and lactation. J Immunol Baltim Md. (1950) 212:513–21. doi: 10.4049/jimmunol.2300560, PMID: 38315948

[149] Dawson CA Pal B Vaillant F Gandolfo LC Liu Z Bleriot C. Tissue-resident ductal macrophages survey the mammary epithelium and facilitate tissue remodelling. Nat Cell Biol. (2020) 22:546–58. doi: 10.1038/s41556-020-0505-0, PMID: 32341550

[150] Gouon-Evans V Rothenberg ME Pollard JW. Postnatal mammary gland development requires macrophages and eosinophils. Dev Camb Engl. (2000) 127:2269–82. doi: 10.1242/dev.127.11.2269, PMID: 10804170

[151] Lee E Hong JJ Samcam Vargas G Sauerwald N Wei Y Hang X. CXCR4+ mammary gland macrophageal niche promotes tumor initiating cell activity and immune suppression during tumorigenesis. Nat Commun. (2025) 16:4854. doi: 10.1038/s41467-025-59972-z, PMID: 40413176 PMC12103607

[152] Zhao L Wang Z Tan Y Ma J Huang W Zhang X. IL-17A/CEBPβ/OPN/LYVE-1 axis inhibits anti-tumor immunity by promoting tumor-associated tissue-resident macrophages. Cell Rep. (2024) 43:115039. doi: 10.1016/j.celrep.2024.115039, PMID: 39643970

[153] Wu S Jiang B Li Z Tang Y Luo L Feng W. Unveiling the key mechanisms of FOLR2+ macrophage-mediated antitumor immunity in breast cancer using integrated single-cell RNA sequencing and bulk RNA sequencing. Breast Cancer Res BCR. (2025) 27:31. doi: 10.1186/s13058-025-01980-4, PMID: 40045365 PMC11881325

[154] Tkach M Thalmensi J Timperi E Gueguen P Névo N Grisard E. Extracellular vesicles from triple negative breast cancer promote pro-inflammatory macrophages associated with better clinical outcome. Proc Natl Acad Sci U.S.A. (2022) 119:e2107394119. doi: 10.1073/pnas.2107394119, PMID: 35439048 PMC9169908

[155] Zhou Y Que KT Zhang Z Yi ZJ Zhao PX You Y. Iron overloaded polarizes macrophage to proinflammation phenotype through ROS/acetyl-p53 pathway. Cancer Med. (2018) 7:4012–22. doi: 10.1002/cam4.1670, PMID: 29989329 PMC6089144

[156] Tan HY Wang N Li S Hong M Wang X Feng Y. The reactive oxygen species in macrophage polarization: reflecting its dual role in progression and treatment of human diseases. Oxid Med Cell Longev. (2016) 2016:2795090. doi: 10.1155/2016/2795090, PMID: 27143992 PMC4837277

[157] Han S Bao X Zou Y Wang L Li Y Yang L. d-lactate modulates M2 tumor-associated macrophages and remodels immunosuppressive tumor microenvironment for hepatocellular carcinoma. Sci Adv. (2023) 9:eadg2697. doi: 10.1126/sciadv.adg2697, PMID: 37467325 PMC10355835

[158] Wang S Liu R Yu Q Dong L Bi Y Liu G. Metabolic reprogramming of macrophages during infections and cancer. Cancer Lett. (2019) 452:14–22. doi: 10.1016/j.canlet.2019.03.015, PMID: 30905817

[159] Waibl Polania J Hoyt-Miggelbrink A Tomaszewski WH Wachsmuth LP Lorrey SJ Wilkinson DS. Antigen presentation by tumor-associated macrophages drives T cells from a progenitor exhaustion state to terminal exhaustion. Immunity. (2025) 58:232–246.e6. doi: 10.1016/j.immuni.2024.11.026, PMID: 39724910

[160] Perez-Diez A Liu X Matzinger P. Neoantigen presentation and IFNγ Signaling on the same tumor-associated macrophage are necessary for CD4 T cell-mediated antitumor activity in mice. Cancer Res Commun. (2022) 2:316–29. doi: 10.1158/2767-9764.CRC-22-0052, PMID: 35903540 PMC9321644

[161] Wang H Yung MMH Ngan HYS Chan KKL Chan DW. The impact of the tumor microenvironment on macrophage polarization in cancer metastatic progression. Int J Mol Sci. (2021) 22:6560. doi: 10.3390/ijms22126560, PMID: 34207286 PMC8235734

[162] Fu C Jiang L Hao S Liu Z Ding S Zhang W. Activation of the IL-4/STAT6 signaling pathway promotes lung cancer progression by increasing M2 myeloid cells. Front Immunol. (2019) 10:2638. doi: 10.3389/fimmu.2019.02638, PMID: 31798581 PMC6863933

[163] Gerloff D Lützkendorf J Moritz RKC Wersig T Mäder K Müller LP. Melanoma-derived exosomal miR-125b-5p educates tumor associated macrophages (TAMs) by targeting lysosomal acid lipase A (LIPA). Cancers. (2020) 12:464. doi: 10.3390/cancers12020464, PMID: 32079286 PMC7072270

[164] Wang S Li J Hong S Wang N Xu S Yang B. Chemotherapy-elicited extracellular vesicle CXCL1 from dying cells promotes triple-negative breast cancer metastasis by activating TAM/PD-L1 signaling. J Exp Clin Cancer Res CR. (2024) 43:121. doi: 10.1186/s13046-024-03050-7, PMID: 38654356 PMC11036662

[165] Zhang Y Zhang X Meng Y Xu X Zuo D. The role of glycolysis and lactate in the induction of tumor-associated macrophages immunosuppressive phenotype. Int Immunopharmacol. (2022) 110:108994. doi: 10.1016/j.intimp.2022.108994, PMID: 35777265

[166] Kes MMG Van den Bossche J Griffioen AW Huijbers EJM. Oncometabolites lactate and succinate drive pro-angiogenic macrophage response in tumors. Biochim Biophys Acta Rev Cancer. (2020) 1874:188427. doi: 10.1016/j.bbcan.2020.188427, PMID: 32961257

[167] Wu JY Huang TW Hsieh YT Wang YF Yen CC Lee GL. Cancer-derived succinate promotes macrophage polarization and cancer metastasis via succinate receptor. Mol Cell. (2020) 77:213–227.e5. doi: 10.1016/j.molcel.2019.10.023, PMID: 31735641

[168] Xiong J Xiao R Zhao J Zhao Q Luo M Li F. Matrix stiffness affects tumor-associated macrophage functional polarization and its potential in tumor therapy. J Transl Med. (2024) 22:85. doi: 10.1186/s12967-023-04810-3, PMID: 38246995 PMC10800063

[169] Jaynes JM Sable R Ronzetti M Bautista W Knotts Z Abisoye-Ogunniyan A. Mannose receptor (CD206) activation in tumor-associated macrophages enhances adaptive and innate antitumor immune responses. Sci Transl Med. (2020) 12:eaax6337. doi: 10.1126/scitranslmed.aax6337, PMID: 32051227 PMC7832040

[170] Cheng H Wang Z Fu L Xu T. Macrophage polarization in the development and progression of ovarian cancers: an overview. Front Oncol. (2019) 9:421. doi: 10.3389/fonc.2019.00421, PMID: 31192126 PMC6540821

[171] Basak U Sarkar T Mukherjee S Chakraborty S Dutta A Dutta S. Tumor-associated macrophages: an effective player of the tumor microenvironment. Front Immunol. (2023) 14:1295257. doi: 10.3389/fimmu.2023.1295257, PMID: 38035101 PMC10687432

[172] Wu P Wu D Zhao L Huang L Chen G Shen G. Inverse role of distinct subsets and distribution of macrophage in lung cancer prognosis: a meta-analysis. Oncotarget. (2016) 7:40451–60. doi: 10.18632/oncotarget.9625, PMID: 27248173 PMC5130019

[173] Yang Z Zhang M Peng R Liu J Wang F Li Y. The prognostic and clinicopathological value of tumor-associated macrophages in patients with colorectal cancer: a systematic review and meta-analysis. Int J Colorectal Dis. (2020) 35:1651–61. doi: 10.1007/s00384-020-03686-9, PMID: 32666290

[174] Zhang J Chang L Zhang X Zhou Z Gao Y. Meta-analysis of the prognostic and clinical value of tumor-associated macrophages in hepatocellular carcinoma. J Investig Surg. (2021) 34:297–306. doi: 10.1080/08941939.2019.1631411, PMID: 31412745

[175] Wang C Lin Y Zhu H Zhou Y Mao F Huang X. The prognostic and clinical value of tumor-associated macrophages in patients with breast cancer: A systematic review and meta-analysis. Front Oncol. (2022) 12:905846. doi: 10.3389/fonc.2022.905846, PMID: 35847911 PMC9280493

[176] Yi B Cheng Y Chang R Zhou W Tang H Gao Y. Prognostic significance of tumor-associated macrophages polarization markers in lung cancer: a pooled analysis of 5105 patients. Biosci Rep. (2023) 43:BSR20221659. doi: 10.1042/BSR20221659, PMID: 36633963 PMC9902841

[177] Yuan X Zhang J Li D Mao Y Mo F Du W. Prognostic significance of tumor-associated macrophages in ovarian cancer: A meta-analysis. Gynecol Oncol. (2017) 147:181–7. doi: 10.1016/j.ygyno.2017.07.007, PMID: 28698008

[178] Majid U Bergsland CH Sveen A Bruun J Eilertsen IA Bækkevold ES. The prognostic effect of tumor-associated macrophages in stage I-III colorectal cancer depends on T cell infiltration. Cell Oncol Dordr Neth. (2024) 47:1267–76. doi: 10.1007/s13402-024-00926-w, PMID: 38407700 PMC11322253

[179] Margul D Yu C AlHilli MM. Tumor immune microenvironment in gynecologic cancers. Cancers. (2023) 15:3849. doi: 10.3390/cancers15153849, PMID: 37568665 PMC10417375

[180] Croci D Santalla Méndez R Temme S Soukup K Fournier N Zomer A. Multispectral fluorine-19 MRI enables longitudinal and noninvasive monitoring of tumor-associated macrophages. Sci Transl Med. (2022) 14:eabo2952. doi: 10.1126/scitranslmed.abo2952, PMID: 36260692

[181] Troiano G Caponio VCA Adipietro I Tepedino M Santoro R Laino L. Prognostic significance of CD68+ and CD163+ tumor associated macrophages in head and neck squamous cell carcinoma: A systematic review and meta-analysis. Oral Oncol. (2019) 93:66–75. doi: 10.1016/j.oraloncology.2019.04.019, PMID: 31109698

[182] Kou Y Li Z Sun Q Yang S Wang Y Hu C. Prognostic value and predictive biomarkers of phenotypes of tumour-associated macrophages in colorectal cancer. Scand J Immunol. (2022) 95:e13137. doi: 10.1111/sji.13137, PMID: 34964155 PMC9286461

[183] Huang X Pan Y Ma J Kang Z Xu X Zhu Y. Prognostic significance of the infiltration of CD163+ macrophages combined with CD66b+ neutrophils in gastric cancer. Cancer Med. (2018) 7:1731–41. doi: 10.1002/cam4.1420, PMID: 29573574 PMC5943426

[184] Dannenmann SR Thielicke J Stöckli M Matter C von Boehmer L Cecconi V. Tumor-associated macrophages subvert T-cell function and correlate with reduced survival in clear cell renal cell carcinoma. Oncoimmunology. (2013) 2:e23562. doi: 10.4161/onci.23562, PMID: 23687622 PMC3655740

[185] Li Z Maeda D Yoshida M Umakoshi M Nanjo H Shiraishi K. The intratumoral distribution influences the prognostic impact of CD68- and CD204-positive macrophages in non-small cell lung cancer. Lung Cancer (2018) 123:127–35. doi: 10.1016/j.lungcan.2018.07.015, PMID: 30089583

[186] Wei J Chen Z Hu M He Z Jiang D Long J. Characterizing intercellular communication of pan-cancer reveals SPP1+ Tumor-associated macrophage expanded in hypoxia and promoting cancer Malignancy through single-cell RNA-seq data. Front Cell Dev Biol. (2021) 9:749210. doi: 10.3389/fcell.2021.749210, PMID: 34676217 PMC8523849

[187] Sun R Han R McCornack C Khan S Tabor GT Chen Y. TREM2 inhibition triggers antitumor cell activity of myeloid cells in glioblastoma. Sci Adv. (2023) 9:eade3559. doi: 10.1126/sciadv.ade3559, PMID: 37172094 PMC10181199

[188] Wang J Zhu N Su X Gao Y Yang R. Novel tumor-associated macrophage populations and subpopulations by single cell RNA sequencing. Front Immunol. (2023) 14:1264774. doi: 10.3389/fimmu.2023.1264774, PMID: 38347955 PMC10859433

[189] Schmid S Csanadi A Kozhuharov N Tchudjin M Kayser C Rawluk J. CC-chemokine ligand 18 is an independent prognostic marker in lymph node-positive non-small cell lung cancer. Anticancer Res. (2018) 38:3913–8. doi: 10.21873/anticanres.12676, PMID: 29970512

[190] Qi X Qi C Wu T Hu Y. CSF1R and HCST: novel candidate biomarkers predicting the response to immunotherapy in non-small cell lung cancer. Technol Cancer Res Treat. (2020) 19:1533033820970663. doi: 10.1177/1533033820970663, PMID: 33153411 PMC7658512

[191] Cao A Yi J Tang X Szeto CW Wu R Wan B. CD47-blocking antibody ZL-1201 promotes tumor-associated macrophage phagocytic activity and enhances the efficacy of the therapeutic antibodies and chemotherapy. Cancer Res Commun. (2022) 2:1404–17. doi: 10.1158/2767-9764.CRC-22-0266, PMID: 36970051 PMC10035405

[192] Beckermann KE Patnaik A Winer I Tan W Bashir B Kyriakopoulos CE. A phase 1b open-label study to evaluate the safety, tolerability, pharmacokinetics, and pharmacodynamics of py314 in combination with pembrolizumab in patients with advanced renal cell carcinoma. Invest New Drugs. (2024) 42:179–84. doi: 10.1007/s10637-024-01419-1, PMID: 38372949

[193] Taylor MH Naing A Powderly J Woodard P Chung L Lin WH. Phase I dose escalation study of IO-108, an anti-LILRB2 antibody, in patients with advanced solid tumors. J Immunother Cancer. (2024) 12:e010006. doi: 10.1136/jitc-2024-010006., PMID: 39567210 PMC11580248

[194] Hong DS Postow M Chmielowski B Sullivan R Patnaik A Cohen EEW. Eganelisib, a first-in-class PI3K-γ Inhibitor, in patients with advanced solid tumors: results of the phase 1/1b MARIO-1 trial. Clin Cancer. (2023) 29:2210–9. doi: 10.1158/1078-0432.CCR-22-3313, PMID: 37000164 PMC10388696

[195] Diab A Ascierto PA Maio M Abdel-Wahab R Negrier S Mortier L. Randomized, open-label, phase III study of tilsotolimod in combination with ipilimumab versus ipilimumab alone in patients with advanced refractory melanoma (ILLUMINATE-301). J Clin Oncol. (2025) 43:1800–9. doi: 10.1200/JCO.24.00727, PMID: 40048691

[196] Reiss KA Angelos MG Dees EC Yuan Y Ueno NT Pohlmann PR. CAR-macrophage therapy for HER2-overexpressing advanced solid tumors: a phase 1 trial. Nat Med. (2025) 31:1171–82. doi: 10.1038/s41591-025-03495-z, PMID: 39920391

[197] Kim AR Yuan Y Ueno NT Johnson ML Gill S Dees EC. A phase 1, first-in-human (FIH) study of the anti-HER2 CAR macrophage CT-0508 in subjects with HER2 overexpressing solid tumors. Alexandria, VA, USA: ASCO. (2022). Available online at: https://www.asco.org/abstracts-presentations/ABSTRACT366030 (Accessed September 15, 2025).

[198] Li X Su X Liu R Pan Y Fang J Cao L. HDAC inhibition potentiates anti-tumor activity of macrophages and enhances anti-PD-L1-mediated tumor suppression. Oncogene. (2021) 40:1836–50. doi: 10.1038/s41388-020-01636-x, PMID: 33564072 PMC7946638

[199] Xu Y Li P Liu Y Xin D Lei W Liang A. Epi-immunotherapy for cancers: rationales of epi-drugs in combination with immunotherapy and advances in clinical trials. Cancer Commun. (2022) 42:493–516. doi: 10.1002/cac2.12313, PMID: 35642676 PMC9198339

[200] Liu M Wang X Du X Zhang Y Ai C Hu-Lieskovan S. CD24Fc ameliorates immune-related adverse events while preserving anti-tumor therapeutic effect. Signal Transduct Target Ther. (2022) 7:224. doi: 10.1038/s41392-022-01030-x, PMID: 35835736 PMC9283527

[201] Yang Y Zhu G Yang L Yang Y. Targeting CD24 as a novel immunotherapy for solid cancers. Cell Commun Signal. (2023) 21:312. doi: 10.1186/s12964-023-01315-w, PMID: 37919766 PMC10623753

[202] Shao C Tang B Chu JCH Lau KM Wong WT Che CM. Macrophage-engaging peptidic bispecific antibodies (pBsAbs) for immunotherapy via a facile bioconjugation strategy. Chem Sci. (2024) 15:11272–8. doi: 10.1039/d4sc00851k, PMID: 39055004 PMC11268508

[203] Wang Z Wang Y He Z Liu C. Emerging cGAS-STING agonist-based nanotherapeutics: mechanistic insights and applications in cancer combination therapy. Adv Sci. (2025) e09890. doi: 10.1002/advs.202509890, PMID: 40842018 PMC12463035

[204] Rodriguez-Garcia A Lynn RC Poussin M Eiva MA Shaw LC O’Connor RS. CAR-T cell-mediated depletion of immunosuppressive tumor-associated macrophages promotes endogenous antitumor immunity and augments adoptive immunotherapy. Nat Commun. (2021) 12:877. doi: 10.1038/s41467-021-20893-2, PMID: 33563975 PMC7873057

[205] Liu L Zhang S Ren Y Wang R Zhang Y Weng S. Macrophage-derived exosomes in cancer: a double-edged sword with therapeutic potential. J Nanobiotechnology. (2025) 23:319. doi: 10.1186/s12951-025-03321-1, PMID: 40287762 PMC12034189

[206] Sun M Bialasek M Mayoux M Lin MS Buck A Marszałek I. Adoptive cell therapy with macrophage-drug conjugates facilitates cytotoxic drug transfer and immune activation in glioblastoma models. Sci Transl Med. (2025) 17:eadr4058. doi: 10.1126/scitranslmed.adr4058, PMID: 40531966

[207] Rannikko JH Hollmén M. Clinical landscape of macrophage-reprogramming cancer immunotherapies. Br J Cancer. (2024) 131:627–40. doi: 10.1038/s41416-024-02715-6, PMID: 38831013 PMC11333586

[208] Sarker D Plummer R Meyer T Sodergren MH Basu B Chee CE. MTL-CEBPA, a small activating RNA therapeutic upregulating C/EBP-α, in patients with advanced liver cancer: A first-in-human, multicenter, open-label, phase I trial. Clin Cancer Res. (2020) 26:3936–46. doi: 10.1158/1078-0432.CCR-20-0414, PMID: 32357963

[209] Li S Zou Y McMasters A Chen F Yan J. Trained immunity: A new player in cancer immunotherapy. eLife. (2025) 14:e104920. doi: 10.7554/eLife.104920, PMID: 40530829 PMC12176388

[210] Chen Q Guo X Ma W. Opportunities and challenges of CD47-targeted therapy in cancer immunotherapy. Oncol Res. (2023) 32:49–60. doi: 10.32604/or.2023.042383, PMID: 38188674 PMC10767231

[211] Sallman DA Al Malki MM Asch AS Wang ES Jurcic JG Bradley TJ. Magrolimab in combination with azacitidine in patients with higher-risk myelodysplastic syndromes: final results of a phase ib study. J Clin Oncol Off J Am Soc Clin Oncol. (2023) 41:2815–26. doi: 10.1200/JCO.22.01794, PMID: 36888930 PMC10414740

[212] ALX Oncology Inc. ALX oncology reports positive interim phase 2 ASPEN-06 clinical trial results of evorpacept for the treatment of advanced HER2-positive gastric cancer . Available online at: https://ir.alxoncology.com/news-releases/news-release-details/alx-oncology-reports-positive-interim-phase-2-aspen-06-clinical/ (Accessed Mar 14,2025).

[213] Movva S Druta M Davis LE Monga V Milhem MM Bailey HH. Safety and clinical activity of TTI-621 in combination with doxorubicin in patients with unresectable or metastatic high-grade leiomyosarcoma: Results from the low-dose expansion cohort. J Clin Oncol. (2023) 41:11508–8. doi: 10.1200/JCO.2023.41.16_suppl.11508

[214] Barkal AA Brewer RE Markovic M Kowarsky M Barkal SA Zaro BW. CD24 signalling through macrophage Siglec-10 is a new target for cancer immunotherapy. Nature. (2019) 572:392–6. doi: 10.1038/s41586-019-1456-0, PMID: 31367043 PMC6697206

[215] Li S Chen D Guo H Yang Y Liu D Yang C. IMM47, a humanized monoclonal antibody that targets CD24, exhibits exceptional anti-tumor efficacy by blocking the CD24/Siglec-10 interaction and can be used as monotherapy or in combination with anti-PD1 antibodies for cancer immunotherapy. Antib Ther. (2023) 6:240–52. doi: 10.1093/abt/tbad020, PMID: 37846296 PMC10576855

[216] Yu J Li S Chen D Guo H Yang Y Liu D. Humanized monoclonal antibody IMM47, targeting CD24, exhibits exceptional anti-tumor efficacy by blocking the CD24/siglec-10 interaction and can be used as monotherapy or in combination with anti-PD1 antibodies for cancer immunotherapy. Blood. (2023) 142:7140. doi: 10.1182/blood-2023-172483 PMC10576855 37846296

[217] Binnewies M Pollack JL Rudolph J Dash S Abushawish M Lee T. Targeting TREM2 on tumor-associated macrophages enhances immunotherapy. Cell Rep. (2021) 37:109844. doi: 10.1016/j.celrep.2021.109844, PMID: 34686340

[218] Umiker B Hashambhoy-Ramsay Y Smith J Rahman T Mueller A Davidson R. Inhibition of LILRB2 by a novel blocking antibody designed to reprogram immunosuppressive macrophages to drive T-cell activation in tumors. Mol Cancer Ther. (2023) 22:471–84. doi: 10.1158/1535-7163.MCT-22-0351, PMID: 36780212

[219] Rolin C Zimmer J Seguin-Devaux C. Bridging the gap with multispecific immune cell engagers in cancer and infectious diseases. Cell Mol Immunol. (2024) 21:643–61. doi: 10.1038/s41423-024-01176-4, PMID: 38789528 PMC11214628

[220] Sewnath CAN Behrens LM van Egmond M. Targeting myeloid cells with bispecific antibodies as novel immunotherapies of cancer. Expert Opin Biol Ther. (2022) 22:983–95. doi: 10.1080/14712598.2022.2098675, PMID: 35854649

[221] Xiong A Wang L Chen J Wu L Liu B Yao J. Ivonescimab versus pembrolizumab for PD-L1-positive non-small cell lung cancer (HARMONi-2): a randomised, double-blind, phase 3 study in China. Lancet. (2025) 405:839–49. doi: 10.1016/S0140-6736(24)02722-3, PMID: 40057343

[222] Wu X Sun Y Yang H Wang J Lou H Li D. Cadonilimab plus platinum-based chemotherapy with or without bevacizumab as first-line treatment for persistent, recurrent, or metastatic cervical cancer (COMPASSION-16): a randomised, double-blind, placebo-controlled phase 3 trial in China. Lancet. (2024) 404:1668–76. doi: 10.1016/S0140-6736(24)02135-4, PMID: 39426385

[223] Malik-Chaudhry HK Prabhakar K Ugamraj HS Boudreau AA Buelow B Dang K. TNB-486 induces potent tumor cell cytotoxicity coupled with low cytokine release in preclinical models of B-NHL. mAbs. (2021) 13:1890411. doi: 10.1080/19420862.2021.1890411, PMID: 33818299 PMC8023237

[224] Thieblemont C Phillips T Ghesquieres H Cheah CY Clausen MR Cunningham D. Epcoritamab, a novel, subcutaneous CD3xCD20 bispecific T-cell-engaging antibody, in relapsed or refractory large B-cell lymphoma: dose expansion in a phase I/II trial. J Clin Oncol. (2023) 41:2238–47. doi: 10.1200/JCO.22.01725, PMID: 36548927 PMC10115554

[225] U.S. Food and Drug Administration. Research C for DE and. FDA approves blinatumomab as consolidation for CD19-positive Philadelphia chromosome-negative B-cell precursor acute lymphoblastic leukemia. Silver Spring, Maryland, USA: FDA (2024). Available online at: https://www.fda.gov/drugs/resources-information-approved-drugs/fda-approves-blinatumomab-consolidation-cd19-positive-philadelphia-chromosome-negative-b-cell (Accessed March 14, 2025).

[226] Shum E Myint H Shaik J Zhou Q Barbu E Morawski A. Clinical benefit through Siglec-15 targeting with NC318 antibody in subjects with Siglec-15 positive advanced solid tumors. J Immunother Cancer. (2021) 9. doi: 10.1136/jitc-2021-SITC2021.490

[227] Ajith A Mamouni K Horuzsko DD Musa A Dzutsev AK Fang JR. Targeting TREM1 augments antitumor T cell immunity by inhibiting myeloid-derived suppressor cells and restraining anti-PD-1 resistance. J Clin Invest. (2023) 133:e167951. doi: 10.1172/JCI167951, PMID: 37651197 PMC10617775

[228] Li J Yang F Wei F Ren X. The role of toll-like receptor 4 in tumor microenvironment. Oncotarget. (2017) 8:66656–67. doi: 10.18632/oncotarget.19105, PMID: 29029545 PMC5630445

[229] Zhou Y Richmond A Yan C. Harnessing the potential of CD40 agonism in cancer therapy. Cytokine Growth Factor Rev. (2024) 75:40–56. doi: 10.1016/j.cytogfr.2023.11.002, PMID: 38102001 PMC10922420

[230] Zhang J Yu S Peng Q Wang P Fang L. Emerging mechanisms and implications of cGAS-STING signaling in cancer immunotherapy strategies. Cancer Biol Med. (2024) 21:45–64. doi: 10.20892/j.issn.2095-3941.2023.0440, PMID: 38172538 PMC10875285

[231] Syntrix Biosystems, Inc. A phase 1, open-label, dose-escalation with expansion study of SX-682 in subjects with metastatic melanoma concurrently treated with pembrolizumab. Bethesda, Maryland, USA: clinicaltrials.gov (2024). Available online at: https://clinicaltrials.gov/study/NCT03161431 (Accessed September 12, 2025).

[232] Tobin RP Davis D Jordan KR McCarter MD. The clinical evidence for targeting human myeloid-derived suppressor cells in cancer patients. J Leukoc Biol. (2017) 102:381–91. doi: 10.1189/jlb.5VMR1016-449R, PMID: 28179538 PMC6608076

[233] Brancewicz J Wójcik N Sarnowska Z Robak J Król M. The multifaceted role of macrophages in biology and diseases. Int J Mol Sci. (2025) 26:2107. doi: 10.3390/ijms26052107, PMID: 40076729 PMC11900619

[234] Song M Liu T Shi C Zhang X Chen X. Bioconjugated manganese dioxide nanoparticles enhance chemotherapy response by priming tumor-associated macrophages toward M1-like phenotype and attenuating tumor hypoxia. ACS Nano. (2016) 10:633–47. doi: 10.1021/acsnano.5b06779, PMID: 26650065 PMC5242343

[235] Shobaki N Sato Y Suzuki Y Okabe N Harashima H. Manipulating the function of tumor-associated macrophages by siRNA-loaded lipid nanoparticles for cancer immunotherapy. J Controlled Release. (2020) 325:235–48. doi: 10.1016/j.jconrel.2020.07.001, PMID: 32649972

[236] Figueiredo P Lepland A Scodeller P Fontana F Torrieri G Tiboni M. Peptide-guided resiquimod-loaded lignin nanoparticles convert tumor-associated macrophages from M2 to M1 phenotype for enhanced chemotherapy. Acta Biomater. (2021) 133:231–43. doi: 10.1016/j.actbio.2020.09.038, PMID: 33011297

[237] Zhang F Parayath NN Ene CI Stephan SB Koehne AL Coon ME. Genetic programming of macrophages to perform anti-tumor functions using targeted mRNA nanocarriers. Nat Commun. (2019) 10:3974. doi: 10.1038/s41467-019-11911-5, PMID: 31481662 PMC6722139

[238] Noorbakhsh Varnosfaderani SM Ebrahimzadeh F Akbari Oryani M Khalili S Almasi F Mosaddeghi Heris R. Potential promising anticancer applications of β-glucans: a review. Biosci Rep. (2024) 44:BSR20231686. doi: 10.1042/BSR20231686, PMID: 38088444 PMC10776902

[239] Li C Song J Guo Z Gong Y Zhang T Huang J. EZH2 inhibitors suppress colorectal cancer by regulating macrophage polarization in the tumor microenvironment. Front Immunol. (2022) 13:857808. doi: 10.3389/fimmu.2022.857808, PMID: 35432300 PMC9010515

[240] Vadevoo SMP Gunassekaran GR Yoo JD Kwon TH Hur K Chae S. Epigenetic therapy reprograms M2-type tumor-associated macrophages into an M1-like phenotype by upregulating miR-7083-5p. Front Immunol. (2022) 13:976196. doi: 10.3389/fimmu.2022.976196, PMID: 36483544 PMC9724234

[241] Liu PS Wang H Li X Chao T Teav T Christen S. α-ketoglutarate orchestrates macrophage activation through metabolic and epigenetic reprogramming. Nat Immunol. (2017) 18:985–94. doi: 10.1038/ni.3796, PMID: 28714978

[242] Ahmed J Stephen B Yang Y Kwiatkowski E Ejezie CL Pant S. Phase ib/II study of lacnotuzumab in combination with spartalizumab in patients with advanced Malignancies. J Immunother Precis Oncol. (2024) 7:73–81. doi: 10.36401/JIPO-23-16, PMID: 38721402 PMC11075470

[243] Sullivan RJ Tsai KK Pavlick AC Buchbinder EI Agarwala SS Ribas A. Millennium pharmaceuticals, inc. An open-label, phase 1b, multi-arm study to evaluate the safety, tolerability, and pharmacodynamics of investigational treatments in combination with standard of care immune checkpoint inhibitors in patients with advanced melanoma. Bethesda, Maryland, USA: National Library of Medicine, clinicaltrials.gov (2024). Available online at: https://clinicaltrials.gov/study/NCT02723006.

[244] Weiss SA Djureinovic D Jessel S Krykbaeva I Zhang L Jilaveanu L. Yale university. A phase I/ib study of APX005M in combination with nivolumab and cabiralizumab in patients with advanced melanoma, non-small cell lung cancer or renal cell carcinoma whose disease has progressed on anti-PD- 1/PD-L1 therapy. Bethesda, Maryland, USA: National Library of Medicine, clinicaltrials.gov (2024). Available online at: https://clinicaltrials.gov/study/NCT03502330.

[245] Xing Q Feng Y Sun H Yang S Sun T Guo X. Scavenger receptor MARCO contributes to macrophage phagocytosis and clearance of tumor cells. Exp Cell Res. (2021) 408:112862. doi: 10.1016/j.yexcr.2021.112862, PMID: 34626585

[246] Gu C Wiest M Zhang W Halder K Zurawski S Zurawski G. Cancer cells promote immune regulatory function of macrophages by upregulating scavenger receptor MARCO expression. J Immunol Baltim Md. (1950) 211:57–70. doi: 10.4049/jimmunol.2300029, PMID: 37212598

[247] La Fleur L Botling J He F Pelicano C Zhou C He C. Targeting MARCO and IL37R on immunosuppressive macrophages in lung cancer blocks regulatory T cells and supports cytotoxic lymphocyte function. Cancer Res. (2021) 81:956–67. doi: 10.1158/0008-5472.CAN-20-1885, PMID: 33293426

[248] Gomez-Roca C Cassier P Zamarin D Machiels JP Perez Gracia JL Stephen Hodi F. Anti-CSF-1R emactuzumab in combination with anti-PD-L1 atezolizumab in advanced solid tumor patients naïve or experienced for immune checkpoint blockade. J Immunother Cancer. (2022) 10:e004076. doi: 10.1136/jitc-2021-004076, PMID: 35577503 PMC9114963

[249] Probst P Simmons R Wall V Zuck M Bouchlaka M Lam S. Abstract 1719: OR2805, an anti-CD163 antibody derived from an elite responder to checkpoint inhibitor therapy relieves immunosuppression caused by tumor associated macrophages. Cancer Res. (2021) 81:1719. doi: 10.1158/1538-7445.AM2021-1719 33472893

[250] Mathiesen H Juul-Madsen K Tramm T Vorup-Jensen T Møller HJ Etzerodt A. Prognostic value of CD163+ macrophages in solid tumor Malignancies: A scoping review. Immunol Lett. (2025) 272:106970. doi: 10.1016/j.imlet.2025.106970, PMID: 39778658

[251] Sánchez-Paulete AR Mateus-Tique J Mollaoglu G Nielsen SR Marks A Lakshmi A. Targeting macrophages with CAR T cells delays solid tumor progression and enhances antitumor immunity. Cancer Immunol Res. (2022) 10:1354–69. doi: 10.1158/2326-6066.CIR-21-1075, PMID: 36095236 PMC10704925

[252] Nandi I Ji L Smith HW Avizonis D Papavasiliou V Lavoie C. Targeting fatty acid oxidation enhances response to HER2-targeted therapy. Nat Commun. (2024) 15:6587. doi: 10.1038/s41467-024-50998-3, PMID: 39097623 PMC11297952

[253] Chen D Barsoumian HB Fischer G Yang L Verma V Younes AI. Combination treatment with radiotherapy and a novel oxidative phosphorylation inhibitor overcomes PD-1 resistance and enhances antitumor immunity. J Immunother Cancer. (2020) 8:e000289. doi: 10.1136/jitc-2019-000289, PMID: 32581056 PMC7319777

[254] Niu M Naguib YW Aldayel AM Shi YC Hursting SD Hersh MA. Biodistribution and *in vivo* activities of tumor-associated macrophage-targeting nanoparticles incorporated with doxorubicin. Mol Pharm. (2014) 11:4425–36. doi: 10.1021/mp500565q, PMID: 25314115 PMC4255729

[255] Tang M Chen B Xia H Pan M Zhao R Zhou J. pH-gated nanoparticles selectively regulate lysosomal function of tumour-associated macrophages for cancer immunotherapy. Nat Commun. (2023) 14:5888. doi: 10.1038/s41467-023-41592-0, PMID: 37735462 PMC10514266

[256] Li Z Ding Y Liu J Wang J Mo F Wang Y. Depletion of tumor associated macrophages enhances local and systemic platelet-mediated anti-PD-1 delivery for post-surgery tumor recurrence treatment. Nat Commun. (2022) 13:1845. doi: 10.1038/s41467-022-29388-0, PMID: 35387972 PMC8987059

[257] Jiang L Qi Y Yang L Miao Y Ren W Liu H. Remodeling the tumor immune microenvironment via siRNA therapy for precision cancer treatment. Asian J Pharm Sci. (2023) 18:100852. doi: 10.1016/j.ajps.2023.100852, PMID: 37920650 PMC10618707

[258] Bi J Liu J Chen X Shi N Wu H Tang H. MiR-155-5p-SOCS1/JAK1/STAT1 participates in hepatic lymphangiogenesis in liver fibrosis and cirrhosis by regulating M1 macrophage polarization. Hum Exp Toxicol. (2023) 42:1–13. Available online at: https://journals.sagepub.com/doi/full/10.1177/09603271221141695?rfr_dat=cr_pub++0pubmed&url_ver=Z39.88-2003&rfr_id=ori%3Arid%3Acrossref.org (Accessed March 18, 2025)., PMID: 36651907 10.1177/09603271221141695

[259] Kimura K Aicher A Niemeyer E Areesawangkit P Tilsed C Fong KP. *In situ* tumor vaccination using lipid nanoparticles to deliver interferon-β mRNA cargo. Vaccines. (2025) 13:178. doi: 10.3390/vaccines13020178, PMID: 40006725 PMC11861666

[260] Hashimoto A Sarker D Reebye V Jarvis S Sodergren MH Kossenkov A. Upregulation of C/EBPα Inhibits suppressive activity of myeloid cells and potentiates antitumor response in mice and patients with cancer. Clin Cancer Res. (2021) 27:5961–78. doi: 10.1158/1078-0432.CCR-21-0986, PMID: 34407972 PMC8756351

[261] Son CJ Carnino JM Lee H Jin Y. Emerging roles of circular RNA in macrophage activation and inflammatory lung responses. Cells. (2024) 13:1407. doi: 10.3390/cells13171407, PMID: 39272979 PMC11394395

[262] Adusei KM Nirschl TR Lee AJ Shen F Wang X Praharaj M. Abstract 170: Targeting of macrophage PI3Kγin prostate cancer using Eganelisib (IPI-549) reprograms immune-suppressive infiltrating macrophages to enhance anti-tumor immune responses and promote immunologically mediated tumor growth. Cancer Res. (2024) 84:170. doi: 10.1158/1538-7445.AM2024-170

[263] Gan L Yang Y Li Q Feng Y Liu T Guo W. Epigenetic regulation of cancer progression by EZH2: from biological insights to therapeutic potential. biomark Res. (2018) 6:10. doi: 10.1186/s40364-018-0122-2, PMID: 29556394 PMC5845366

[264] Morel KL Sheahan AV Burkhart DL Baca SC Boufaied N Liu Y. EZH2 inhibition activates a dsRNA–STING–interferon stress axis that potentiates response to PD-1 checkpoint blockade in prostate cancer. Nat Cancer. (2021) 2:444–56. doi: 10.1038/s43018-021-00185-w, PMID: 33899001 PMC8061902

[265] Kim HJ Cantor H Cosmopoulos K. Overcoming immune checkpoint blockade resistance via EZH2 inhibition. Trends Immunol. (2020) 41:948–63. doi: 10.1016/j.it.2020.08.010, PMID: 32976740

[266] Zhang Y Chen J Liu H Mi R Huang R Li X. The role of histone methylase and demethylase in antitumor immunity: A new direction for immunotherapy. Front Immunol. (2023) 13:1099892. doi: 10.3389/fimmu.2022.1099892, PMID: 36713412 PMC9874864

[267] Gatchalian J Liao J Maxwell MB Hargreaves DC. Control of stimulus-dependent responses in macrophages by SWI/SNF chromatin remodeling complexes. Trends Immunol. (2020) 41:126–40. doi: 10.1016/j.it.2019.12.002, PMID: 31928914 PMC6995420

[268] Buffen K Oosting M Quintin J Ng A Kleinnijenhuis J Kumar V. Autophagy controls BCG-induced trained immunity and the response to intravesical BCG therapy for bladder cancer. PloS Pathog. (2014) 10:e1004485. doi: 10.1371/journal.ppat.1004485, PMID: 25356988 PMC4214925

[269] Covarrubias AJ Aksoylar HI Horng T. Control of macrophage metabolism and activation by mTOR and Akt signaling. Semin Immunol. (2015) 27:286–96. doi: 10.1016/j.smim.2015.08.001, PMID: 26360589 PMC4682888

[270] Bhat MF Srdanović S Sundberg LR Einarsdóttir HK Marjomäki V Dekker FJ. Impact of HDAC inhibitors on macrophage polarization to enhance innate immunity against infections. Drug Discov Today. (2024) 29:104193. doi: 10.1016/j.drudis.2024.104193, PMID: 39332483

[271] Krushkal J Zhao Y Roney K Zhu W Brooks A Wilsker D. Association of changes in expression of HDAC and SIRT genes after drug treatment with cancer cell line sensitivity to kinase inhibitors. Epigenetics. (2024) 19:2309824. doi: 10.1080/15592294.2024.2309824, PMID: 38369747 PMC10878021

[272] Klichinsky M Ruella M Shestova O Lu XM Best A Zeeman M. Human chimeric antigen receptor macrophages for cancer immunotherapy. Nat Biotechnol. (2020) 38:947–53. doi: 10.1038/s41587-020-0462-y, PMID: 32361713 PMC7883632

[273] Feng F Shen J Qi Q Zhang Y Ni S. Empowering brain tumor management: chimeric antigen receptor macrophage therapy. Theranostics. (2024) 14:5725–42. doi: 10.7150/thno.98290, PMID: 39310093 PMC11413779

[274] Annunziata CM Ghobadi A Pennella EJ Vanas J Powell C Pavelova M. Feasibility and preliminary safety and efficacy of first-in-human intraperitoneal delivery of MCY-M11, anti-human-mesothelin CAR mRNA transfected into peripheral blood mononuclear cells, for ovarian cancer and Malignant peritoneal mesothelioma. J Clin Oncol. (2020) 38:3014–4. doi: 10.1200/JCO.2020.38.15_suppl.3014

[275] CellOrigin. CellOrigin announced treatment of the first patient with CAR-M in China and reported the second generation of CAR-M for solid tumors . Available online at: https://www.prnewswire.com/news-releases/cellorigin-announced-treatment-of-the-first-patient-with-car-m-in-China-and-reported-the-second-generation-of-car-m-for-solid-tumors-302020068.html (Accessed March 14, 2025).

[276] Myeloid therapeutics. A phase 1/2, open-label, first-in-human, multiple ascending dose multicenter study of MT-101 in subjects with CD5+ Relapsed/refractory T cell lymphoma. Bethesda, Maryland, USA: clinicaltrials.gov (2023). Available online at: https://clinicaltrials.gov/study/NCT05138458 (Accessed March 14, 2025).

[277] Brempelis KJ Cowan CM Kreuser SA Labadie KP Prieskorn BM Lieberman NAP. Genetically engineered macrophages persist in solid tumors and locally deliver therapeutic proteins to activate immune responses. J Immunother Cancer. (2020) 8:e001356. doi: 10.1136/jitc-2020-001356, PMID: 33115946 PMC7594542

[278] Li J Chen P Ma W. The next frontier in immunotherapy: potential and challenges of CAR-macrophages. Exp Hematol Oncol. (2024) 13:76. doi: 10.1186/s40164-024-00549-9, PMID: 39103972 PMC11302330

[279] Zhu Q Huang X Deng B Guan L Zhou H Shi B. Tumor micro-environment induced TRAIL secretion from engineered macrophages for anti-tumor therapy. Cell Immunol. (2024) 403–404:104857. doi: 10.1016/j.cellimm.2024.104857, PMID: 39032210

[280] Tanito K Nii T Yokoyama Y Oishi H Shibata M Hijii S. Engineered macrophages acting as a trigger to induce inflammation only in tumor tissues. J Controlled Release. (2023) 361:885–95. doi: 10.1016/j.jconrel.2023.04.010, PMID: 37080897

[281] Miao C Huang G Wen Y He Y Bai P Yan W. M1 macrophage-derived extracellular vesicles loaded with CX3CR1 siRNA for the treatment of pancreatic cancer. ACS Appl Mater Interfaces. (2025) 17:40226–36. doi: 10.1021/acsami.5c07950, PMID: 40600720

[282] Liu H Ouyang Z Li S. Advances of M1 macrophages-derived extracellular vesicles in tumor therapy. BioMed Pharmacother. (2024) 181:117735. doi: 10.1016/j.biopha.2024.117735, PMID: 39644871

[283] Lecoultre M Walker PR El Helali A. Oncolytic virus and tumor-associated macrophage interactions in cancer immunotherapy. Clin Exp Med. (2024) 24:202. doi: 10.1007/s10238-024-01443-8, PMID: 39196415 PMC11358230

[284] Taciak B Białasek M Kucharzewska-Siembieda P Kiraga Ł Szulc A Górka E. Abstract B24: The macrophage-drug conjugate (MDC) as a “Trojan horse” approach in cancer therapy. Cancer Immunol Res. (2020) 8:B24. doi: 10.1158/2326-6074.TUMIMM18-B24

[285] Taciak B Bialasek M Kubiak M Marszalek I Gorczak M Osadchuk O. Harnessing macrophage-drug conjugates for allogeneic cell-based therapy of solid tumors via the TRAIN mechanism. Nat Commun. (2025) 16:1327. doi: 10.1038/s41467-025-56637-9, PMID: 39900573 PMC11790938

[286] Bialasek M Sun M Marszalek I Taciak B Weller M Rygiel T. Abstract A037: macrophage-ferritin-drug conjugates: A novel approach to overcome glioblastoma drug resistance and induce long-term tumor immunity. Cancer Immunol Res. (2025) 13:A037. doi: 10.1158/2326-6074.IO2025-A037

[287] Habib S Osborn G Willsmore Z Chew MW Jakubow S Fitzpatrick A. Tumor associated macrophages as key contributors and targets in current and future therapies for melanoma. Expert Rev Clin Immunol. (2024) 20:895–911. doi: 10.1080/1744666X.2024.2326626, PMID: 38533720 PMC11286214

[288] Nielsen MC Andersen MN Rittig N Rødgaard-Hansen S Grønbæk H Moestrup SK. The macrophage-related biomarkers sCD163 and sCD206 are released by different shedding mechanisms. J Leukoc Biol. (2019) 106:1129–38. doi: 10.1002/JLB.3A1218-500R, PMID: 31242338

[289] Wahner A. CD47 levels are prognostic of response to magrolimab plus docetaxel in metastatic NSCLC. Monroe, New Jersey, USA: OncLive, MJH Life Sciences (2025). Available online at: https://www.onclive.com/view/cd47-levels-are-prognostic-of-response-to-magrolimab-plus-docetaxel-in-metastatic-nsclc.

[290] Zhai T Mitamura T Wang L Kubota SI Murakami M Tanaka S. Combination therapy with bevacizumab and a CCR2 inhibitor for human ovarian cancer: An *in vivo* validation study. Cancer Med. (2023) 12:9697–708. doi: 10.1002/cam4.5674, PMID: 36810973 PMC10166889

[291] Ouyang P Wang L Wu J Tian Y Chen C Li D. Overcoming cold tumors: a combination strategy of immune checkpoint inhibitors. Front Immunol. (2024) 15:1344272. doi: 10.3389/fimmu.2024.1344272, PMID: 38545114 PMC10965539

[292] Wang AX Ong XJ D’Souza C Neeson PJ Zhu JJ. Combining chemotherapy with CAR-T cell therapy in treating solid tumors(2023). 10.3389/fimmu.2023.1140541 PMC10026332 36949946

